# CACCT: An Automated Tool of Detecting Complicated Cardiac Malformations in Mouse Models

**DOI:** 10.1002/advs.201903592

**Published:** 2020-02-20

**Authors:** Qing Chu, Haobin Jiang, Libo Zhang, Dekun Zhu, Qianqian Yin, Hao Zhang, Bin Zhou, Wenzhang Zhou, Zhang Yue, Hong Lian, Lihui Liu, Yu Nie, Shengshou Hu

**Affiliations:** ^1^ State Key Laboratory of Cardiovascular Disease Fuwai Hospital National Center for Cardiovascular Disease Chinese Academy of Medical Sciences and Peking Union Medical College Beijing 100037 China; ^2^ State Key Laboratory of Computer Science Institute of Software Chinese Academy of Sciences Beijing 100089 China; ^3^ Heart Center and Shanghai Institution of Pediatric Congenital Heart Diseases Shanghai Children's Medical Center National Children's Medical Center Shanghai Jiao Tong University School of Medicine Shanghai 200127 China; ^4^ State Key Laboratory of Cell Biology CAS Center for Excellence in Molecular Cell Science Shanghai Institute of Biochemistry and Cell Biology Chinese Academy of Sciences (CAS) University of Chinese Academy of Sciences Shanghai 200031 China; ^5^ Department of Cardiovascular Surgery Union Hospital Tongji Medical College Huazhong University of Science and Technology Wuhan 430022 China

**Keywords:** computer‐assisted diagnosis, congenital heart disease, double‐outlet right ventricle, micro‐CT, ventricular septal defects

## Abstract

Congenital heart disease (CHD) is the major cause of morbidity/mortality in infancy and childhood. Using a mouse model to uncover the mechanism of CHD is essential to understand its pathogenesis. However, conventional 2D phenotyping methods cannot comprehensively exhibit and accurately distinguish various 3D cardiac malformations for the complicated structure of heart cavity. Here, a new automated tool based on microcomputed tomography (micro‐CT) image data sets known as computer‐assisted cardiac cavity tracking (CACCT) is presented, which can detect the connections between cardiac cavities and identify complicated cardiac malformations in mouse hearts automatically. With CACCT, researchers, even those without expert training or diagnostic experience of CHD, can identify complicated cardiac malformations in mice conveniently and precisely, including transposition of the great arteries, double‐outlet right ventricle and atypical ventricular septal defect, whose accuracy is equivalent to senior fetal cardiologists. CACCT provides an effective approach to accurately identify heterogeneous cardiac malformations, which will facilitate the mechanistic studies into CHD and heart development.

## Graphical abstract

An automated tool based on microcomputed tomography (CT) images known as computer‐assisted cardiac cavity tracking (CACCT) is established to identify complicated cardiac malformations in mice. With CACCT, researchers, even those without expert training or diagnostic experience of congenital heart diseases, can identify complicated cardiac malformations effectively, including transposition of the great arteries, double‐outlet right ventricle and atypical ventricular septal defect, whose accuracy is equivalent to senior fetal cardiologists.

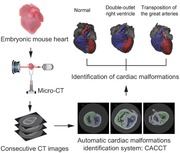

## 1 Introduction

As the major cause of morbidity/mortality in infancy and childhood,^[^ 1, 2 ^]^ 1.4 million congenital heart diseases (CHD) are diagnosed every year worldwide,^[^ 3 ^]^ with a frequency of 0.8–1.2% among live births.^[^ 3, 4 ^]^ Uncovering the underlying molecular mechanisms of CHD is crucial for developing effective prevention and intervention strategies. Mouse model is ideal for investigating CHD genetics, developmental etiology and mechanisms. However, for CHD and heart development researchers, phenotyping mouse cardiac malformations remains challenging.

The process of phenotyping cardiac structural malformations includes two steps: 1) identifying cardiac cavities; 2) determining the connections of the cavities. Taking transposition of the great arteries (TGA), a complicated outflow tract (OFT) malformation, as an example, the characterization of TGA is right ventricle (RV) connects to aorta and left ventricle (LV) inversely to pulmonary artery (PA), therefore the essence of identifying TGA is to locate ventricles and the great arteries and determine their connections.

Hematoxylin & Eosin (HE) staining and ultrasound biomicroscopy (UBM) are widely utilized to identify mouse heart structural defects due to their low cost.^[^ 5, 6, 7 ^]^ However, it is difficult to obtain proper sections in HE staining or acoustic window in UBM to show diagnosis‐required cavities and judge the connecting relationship to identify heart structural defects,^[^ 5, 7, 8 ^]^ because obtaining the proper sections needs long‐term training and is difficult to avoid artificial errors.

Microcomputed tomography (micro‐CT) can provide consecutive whole heart image datasets, comprising hundreds to thousands images depending on the size of samples and scanning pixel.^[^ 9, 10, 11 ^]^ With 3D heart reconstructions of CT images, researchers can observe the sections of a heart at any angle, which is beneficial to analyze intracardiac structures and identify heart structural defects theoretically. However, using plane CT images is still hard to interpret the various and 3D defective connections of cavities in cardiac malformations, which limited the application of CT in mouse cardiac malformation phenotyping.

Here, we established an automated method to identify cardiac structural defects, known as computer‐assisted cardiac cavity tracking system (CACCT), which can detect targeted cardiac cavities in hundreds of consecutive CT images and judge the connecting relationship between the detected cavities automatically. By running CACCT, researchers, even without diagnostic experience, can identify and distinguish complicated cardiac structural defects within 5 min. We believe that CACCT is a powerful tool to construct precise links between genotype and cardiac phenotypes and drive the development of CHD studies.

## 2 Results

### 2.1 Establishment of CACCT

**Figure 1:**
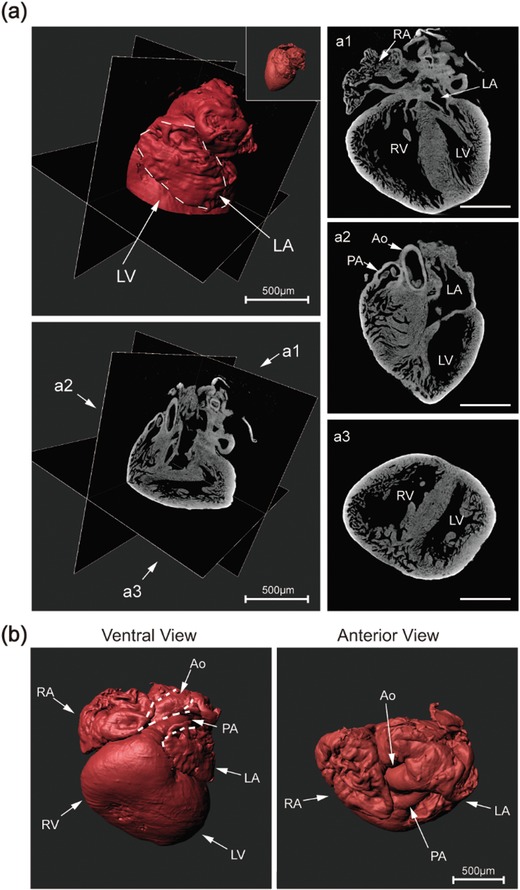
CT images cannot show 3D connections of cavities. a) 3D reconstruction data of cardiac CT images shows intracardiac structure in obtain plane sections at arbitrary angles. Right column indicate three sections of CT images at different angle. The insets show 3D myocardium image. RV, right ventricle; LV, left ventricle; RA, right atrium; LA, left atrium.; Ao, aorta; PA, pulmonary artery. Scale bar: 500 µm. b) 3D reconstruction of cardiac CT images shows outward appearance of ventricles, atrium, and great vessels.

We collected hearts from fetal mice at embryonic day 17.5 (E17.5), scanned the hearts by high‐resolution micro‐CT and obtained 990–1050 consecutive CT images (2.3–3.2 µm voxel size) from each heart. In the CT images, we observed the main cardiac chambers, including ventricles, atria, aorta, and pulmonary artery (**Figure** **1**a). With 3D reconstruction of CT consecutive images, we acquired 3D myocardium images showed outward appearance of ventricles, atria and great vessels (Figure 1b), which provided us with the position of the chambers. We could obtain plane sections at arbitrary angles in 3D reconstruction data, facilitating the intracardiac structure observation (Figure 1a). However, it is hard to track connection of cardiac cavities, neither using 3D myocardium images nor plane sections. We binarized and inverted the micro‐CT to obtain the volume information from the cavity of the heart (Figure S1a,b,c, Supporting Information); and found that inversion of CT images could not segment ventricles and the great arteries (Figure S1d,e, Supporting Information).

**Figure 2:**
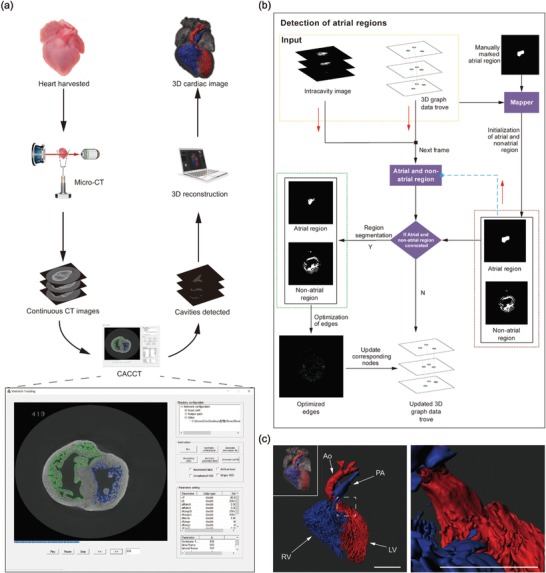
CACCT enables automatic 3D cardiac cavity tracking. a) The CACCT pipeline. Computer‐assisted detection includes automated tracking cardiac cavities by self‐developed software: cavity tracker. 3D reconstruction can be achieved using commonly available 3D reconstructing software, such as Imaris. b) The atrial cavity detection strategy. N, no. c) 3D ventricular and great arterial cavity images generated by CACCT. The connected ventricle and great artery are indicated with the same color. Blue indicates the right ventricular cavities and pulmonary artery; Red indicates the left ventricular cavities and aorta. The insets show 3D myocardium and cardiac cavity images. RV, right ventricle; LV, left ventricle; Ao, aorta; PA, pulmonary artery. Scale bar: 500 µm.

To track the connection of cardiac cavities in 3D level, we established the computer‐assisted cardiac cavity tracking (CACCT) system (**Figure** **2**a). CACCT captured the region of interest that represented the cardiac cavities in each CT image. Then, CACCT made full use of the relationship between frames to build a 3D graphical data trove. Finally, basing on the 3D graphical data trove, CACCT could detect and track cardiac cavities, section by section in consecutive CT image data sets, and determine the connections of the detected cavities (Figures 2b and 4d). The principle that CACCT detect cardiac malformations was to determine whether the CACCT‐detected connection of cavities accord with cardiac anatomy: PA connects to RV and aorta to LV; otherwise, it was indicated that there were OFT associated malformations (**Table** **1**). CACCT was able to process raw data with different resolution. We uniformly used CT images with resolution of 992 × 1014 and smallest detectable area of CACCT was 10 pixel.

**Table 1:**
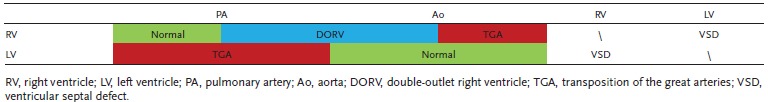
Phenotypes corresponding to connecting relationship between cardiac cavities

We wonder if CACCT could be used to identify OFT structure. First, we need to figure out whether CACCT could identify normal OFT. We run CACCT on 11 normal E17.5 mouse hearts and found CACCT could automatically track pathway from ventricles to the great arteries in consecutive CT images data sets and automatedly identified the tested hearts as normal phenotype with 100% accuracy (*n* = 11/11). CACCT also detected and excluded atria to avoid disturbance of atria during analyzing OFT structure (Figure 2b; Movie S1, Supporting Information). We obtained 3D OFT images by 3D reconstruction of CACCT‐detected OFT (Figure 2c; Movie S2, Supporting Information). We confirmed that pulmonary artery connected to RV and aorta to LV in the 3D images of all 11 tested hearts, which indicated that CACCT could accurately track OFT in consecutive image data sets. Compared to 2D images obtained by conventional methods, CACCT‐generated 3D OFT images provided accurate phenotyping reports and general insight into cavity morphology and the route of blood flow without requirement of burdensome image analyses and diagnostic knowledge.

To evaluate the accuracy of CACCT segmentation, we inspected the segmenting results of CACCT section by section and marked error regions larger than 15 × 15 µm. The precision and sensitivity were both higher than 99.77% in normal hearts (**Table** **2**), which indicated that CACCT could accurately detect ventricular chambers in normal hearts. We found that the coefficient of variation (CV) of precision and sensitivity were lower than 0.38% (Table 2), which indicated that CACCT segmentation was stable among different normal hearts.

**Table 2:** Accuracy and stability of CACCT segmentation

	Left ventricle		Right ventricle	
	Precision (CV)	Sensitivity (CV)	Precision (CV)	Sensitivity (CV)
Normal hearts (*n* = 11)	99.91% (0.23%)	99.92% (0.13%)	99.98% (0.06%)	99.77% (0.38%)
RA‐treated hearts (*n* = 22)	99.85% (0.63%)	99.90% (0.15%)	99.88% (0.24%)	99.87% (0.30%)

CV, coefficient of variance; RA, retinoic acid.

**Table 3:** Clinical and CACCT diagnosis in blinded analyses of outflow tract phenotype

Subject NO.	Clinical Diagnosis	CACCT Diagnosis	Comparison with Clinical Diagnosis
1	TGA	TGA	Agree
2	Normal	Normal	Agree
3	TGA	TGA	Agree
4	TGA	TGA	Agree
5	Normal	Normal	Agree
6	DORV	DORV	Agree
7	TGA	TGA	Agree
8	Normal	Normal	Agree
9	TGA	TGA	Agree
10	Normal	Normal	Agree
11	TGA	TGA	Agree
12	DORV	DORV	Agree
13	DORV	DORV	Agree
14	Normal	Normal	Agree
15	TGA	TGA	Agree
16	DORV	DORV	Agree
17	TGA	TGA	Agree
18	TGA	TGA	Agree
19	Normal	Normal	Agree
20	DORV	DORV	Agree
21	TGA	TGA	Agree
22	Normal	Normal	Agree
23	TGA	TGA	Agree
24	TGA	TGA	Agree
25	DORV	DORV	Agree
26	TGA	TGA	Agree
27	DORV	DORV	Agree
28	Normal	Normal	Agree
29	TGA	TGA	Agree
30	Normal	Normal	Agree
31	Normal	Normal	Agree
32	Normal	Normal	Agree
33	DORV	DORV	Agree

TGA, transposition of the great arteries; DORV, double‐outlet right ventricle.

In conclusion, based on CT images of normal mouse hearts, we demonstrated that CACCT enables automatically tracking cardiac cavities and judgement on the connecting relationship and spatial extend direction of detected cavities, which is believed to be potential for identification of OFT malformations.

### 2.2 CACCT is Effective to Identify Multiple Murine Cardiac Malformations

**Figure 3:**
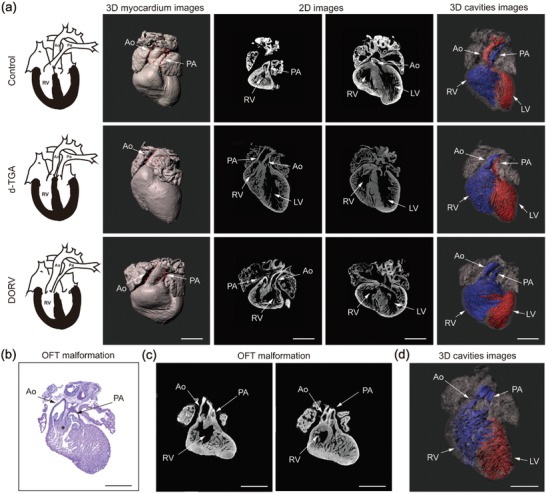
CACCT identifies OFT malformations. a) Upper row: normal alignment of the aorta (Ao) and pulmonary artery (PA) in control embryonic mice. RV, right ventricle; LV, left ventricle. The 3D myocardium images were generated by direct reconstruction of the CT dataset and show only the appearance of the heart. The 3D cavity images were acquired by CACCT with automatic cavity detection and show the connecting relationships between the ventricles and great arteries. Middle and bottom rows: subsets of misalignment of the aorta and pulmonary artery, including dextro‐transposition of the great arteries (d‐TGA) and double‐outlet right ventricle (DORV) with a VSD. b) HE staining of an E17.5 murine heart with an RA‐treated OFT malformation, with Ao and PA misalignment; whether the cavity connected to the PA is the RV or LV is unclear. The asterisk denotes the myocardium tissue beneath the PA and Ao; it is difficult to determine whether the myocardium tissue is a ventricular septum. c) Sections of the CT image dataset from RA‐treated hearts showing misalignment of the Ao and PA. The left graph shows the Ao connecting to the RV; it is difficult to determine whether the cavity connected to the PA is the RV or LV. d) A 3D cavity image generated by CACCT of the heart presented in (c), showing a DORV phenotype. Scale bar: 500 µm.

To explore whether CACCT could detect cardiac structural defects, we induced CHD in mice on E8.5 with all‐trans retinoic acid (RA), which was reported to induce multiple cardiac malformations.^[^ 8, 12 ^]^ We scanned the RA‐treated embryonic hearts (E17.5; *n* = 22) by micro‐CT and obtained ≈1000 consecutive CT images each embryonic heart. Then we applied CACCT to analyze the presence of cardiac malformations in RA‐treated mice by tracking the connection between cardiac cavities and reconstruct the CACCT‐detected cardiac cavities at 3D level. CACCT automatic analyses showed that all 22 RA‐treated hearts suffered with heart structural defects. Fourteen of them were identified as TGA by CACCT, because CACCT found the aorta originated from the RV and the pulmonary artery from the LV (**Figure** **3**a; Movies S3 and S4, Supporting Information). The other eight RA‐treated hearts were diagnosed as double‐outlet right ventricle (DORV) by CACCT, because CACCT detected the RV connected to both aorta and pulmonary artery (Figure 3a; Movies S4–S6, Supporting Information). It was difficult to distinguish TGA from DORV using HE slides, because plane images of HE could hardly show the accurate connecting relationship between ventricles and the great arteries (Figure 3b,c,d).

**Figure 4:**
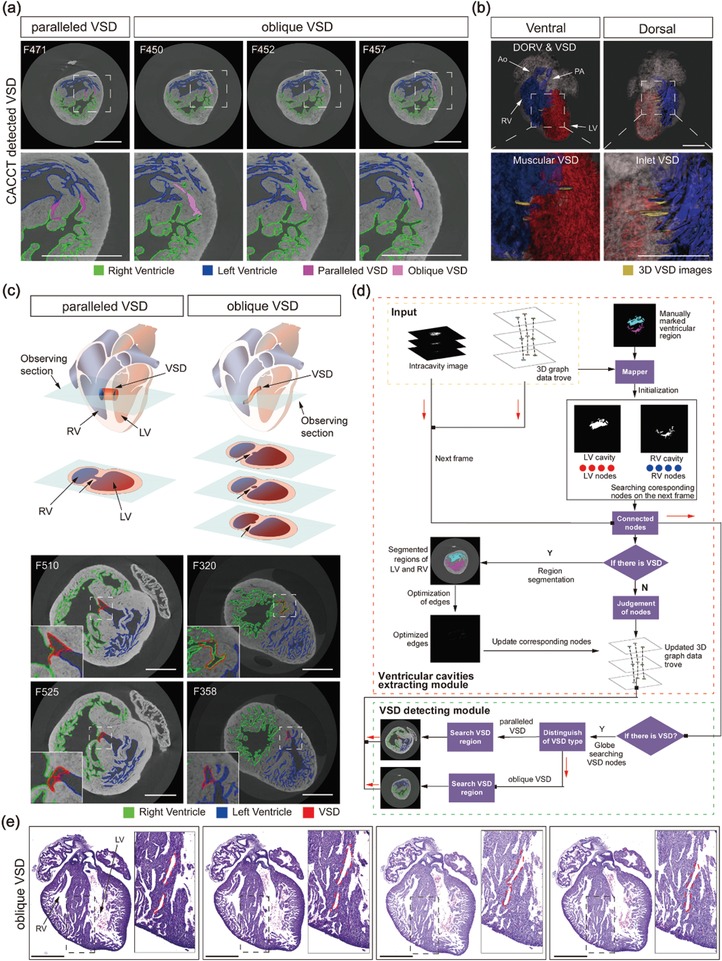
CACCT detects VSD. a) CACCT‐detected morphology and position of paralleled and oblique VSD. b) 3D cardiac cavity images generated by CACCT, with VSDs viewed from the ventral and dorsal sides. c) Schematic diagram and transverse section of CT images showing paralleled and oblique VSD in RA‐treated hearts. The type of VSD was defined depending on the characteristics of the consecutive CT image dataset. For paralleled VSD, the left and right ventricles were connected on one frame during inference of the cardiac cavity [frame (F) 510; left column]; oblique VSD obliquely intersects the transverse section. The VSD connected with the RV on F320 and with the LV on F358 (right column). RV, right ventricle; LV, left ventricle; short arrow indicates VSD. d) The ventricular and great arterial cavity detection strategy and VSD‐detecting module. RV, right ventricle; LV, left ventricle; Y, yes; N, no. e) Whole‐mount consecutive HE staining of E17.5 murine hearts showing RA‐treated oblique VSD. The VSD connected to the right ventricle in the left graph gradually connects to the left ventricle across consecutive slides. Scale bar: 500 µm.

Simultaneously, in 22 RA‐treated hearts, the linkage between LV and RV were detected and diagnosed as ventricular septal defect (VSD). CACCT also provided information on the spatial position and the shape of these VSD channels in consecutive CT images (**Figure** **4**a; Movie S7, Supporting Information). The information of VSD channels, provided by CACCT, indicated that there were supracristal, paramembranous, inlet, and muscular VSDs in RA‐treated hearts (Figure 4b). Only supracristal and paramembranous VSDs have been reported in RA‐treated hearts previously by HE staining analyses.^[^ 5, 12, 13 ^]^ Two types of VSD were identified by CACCT: 1) paralleled VSD channel: a channel parallel to observing sections, which connected LV and RV in one frame (Figure 4c); 2) oblique VSD channel: a channel obliquely intersected the observing section, which connected with one or no ventricular chambers in one image (Figure 4c). It is difficult to detect the oblique VSD channels not only in observation of consecutive CT images, but also in consecutive HE staining images, due to the multiple spatial angles of these VSD channels (Figure 4e).

The precision and sensitivity were both higher than 99.85% and the CV of precision and sensitivity in RA‐treated hearts were lower than 0.63% in RA‐treated hearts (*n* = 22, Table 2), which indicated that CACCT segmentation was accurate and reproducible among different RA‐treated hearts. The analyses of CACCT with nearly 1000 CT sections can be completed within 5 min, and can make a one‐time diagnosis of TGA, DORV and various VSDs. The related process does not require the participation of researchers with experience in CHD diagnosis and avoids artificial error.

### 2.3 CACCT Makes Heart Structural Analyses Precise and Convenient

To test the accuracy of CACCT for malformation identification, two senior fetal cardiologists (S.H. and H.Z.), with more than 20‐year experience of CHD surgery, were asked to identify the cardiac phenotypes basing on primary consecutive CT images of normal heart (*n* = 11) and RA‐treated hearts (*n* = 22). The results of the two senior fetal cardiologists are consistent. Senior fetal cardiologists identified OFT malformations and distinguished TGA from DORV in primary consecutive CT images. The identification results of TGA and DORV by CACCT were consistent with those by cardiologists **Table** **3**. The diagnostic ability of TGA and DORV by CACCT is equivalent to that by senior fetal cardiologists.

On the other hand, we want to know whether it is feasible to tacking cardiac cavities by manually marking CT images. We marked the contour of ventricular chamber and the related great artery section by section (Details were described in the Supporting Information; Movie S8, Supporting Information). The process of manually CT image cardiac cavity marking should take 29.90 ± 1.33 h per heart, whose time cost is over 380 times comparing to CACCT (4.80 ± 0.84 min per heart; *p* < 0.0001). Furthermore, manual marking required the operators with rich anatomical knowledge and operational experience. Notably, manual marking is easy to neglect small cavities, which brought artificial errors in cavity detection (Figure S1f and Movie S9, Supporting Information). Therefore, manually marking cardiac cavities in consecutive CT images was not a feasible method for identification of cardiac malformations.

Thus, CACCT is a feasible tool to analyze heart structure and identify cardiac malformations, which is equivalent to a senior fetal cardiologist and more convenient.

## 3 Discussion

Efficient and accurate phenotypic identification of murine cardiac structural malformations is the basis to reveal the pathogenesis of CHD using mouse model. The widely used methods for identifying mouse CHD were HE staining and UBM,^[^ 5, 6, 7 ^]^ which require researchers with rich diagnostic experience and are hard to avoid artificial errors. Here we developed an automated 3D‐image analysis system known as CACCT that performs automatic 3D cardiac cavity tracking to monitor and map the connecting relationships of cardiac cavities (Figure 2a). CACCT can effectively identify TGA, DORV, and VSD at one time, whose phenotyping accuracy is equivalent to senior fetal cardiologists. With the assistance of CACCT, researchers, even those without experience in CHD diagnosis, can accurately identify complicated cardiac malformations. All imaging and image processing in this study was generated from standard micro‐CT sequences, self‐developed software and nonmodified computer hardware.

CACCT can detect oblique VSD channel, which is hard be identified by HE staining. HE staining can only be used to identify VSD channels paralleled to the slide, which connected LV and RV in the slide. However, in fact the direction of VSD is 3D, thus it is prone to misdiagnose VSD channels obliquely intersected at HE slide (Figure 4e). Xie et al. combined atlas‐based segmentation and snake evolution technique to detect VSD in murine hearts.^[^ 14 ^]^ However, the method can only recognize paralleled VSD displaying in single section. Basing on 3D graphical data trove and graph iterative techniques, CACCT can detect both paralleled and oblique VSDs in consecutive image data sets. The detection of oblique VSDs by CACCT will promote studies into pathogenesis of the kind of VSD.

Some previous methods of image processing and machine learning can segment organs and tissues in medical images effectively, such as level set, active contour, and appearance model.^[^ 15, 16, 17, 18 ^]^ Compared with traditional algorithms, deep learning, such as U‐Net,^[^ 19 ^]^ Attention U‐Net,^[^ 20 ^]^ and Dense‐Net,^[^ 21 ^]^ directly learns the characteristics of the target from a large amount of data through end‐to‐end network. With these features, methods based on deep learning achieve high accuracy in organ and tissue segmentation. The above methods can only be used in 2D, but it cannot work in 3D.

In summary, we developed an automatic 3D cardiac cavity tracking system known as CACCT, which help researchers, even those without CHD‐diagnostic experience, identify kinds of complicated cardiac malformations effectively. Importantly, we believe that CACCT will help establish more precise links between murine genotypes and cardiac phenotypes and rejuvenate research into heart development and CHD pathogenesis.

## 4 Experimental Section

##### Animals

All animals were used according to guidelines of the Institutional Animal Care and Use Committee of the Fuwai Hospital, National Center for Cardiovascular Disease, Chinese Academy of Medical Sciences and Peking Union Medical College. C57BL/J mice (8–10 weeks‐of‐age) were kept under a 12‐h light–dark cycle in specific‐pathogen‐free conditions. Females were checked by vaginal smear in the evening (between 6:00 and 7:00 pm) and placed with males when in proestrus. The morning after mating, the females were checked for vaginal plugs, and the plug day was defined as embryonic day 0.5 (E0.5). All‐trans retinoic acid (RA) (R2625, Sigma‐Aldrich, USA) was first dissolved in dimethyl sulfoxide (100 mg mL^−1^) (D2650, Sigma‐Aldrich, USA) and stored at −20 °C. The solution was dissolved in corn oil (10 mg mL^−1^) (D8267, Sigma‐Aldrich, USA) to make gavage solution. At E8.5, pregnant mice were administered RA (50 mg kg^−1^) by oral gavage to induce OFT malformation.^[^ 12 ^]^ The control pregnant mice were administered dimethyl sulfoxide dissolved in corn oil by oral gavage at E8.5.

##### Histology

Histology was performed as previously described.^[^ 22 ^]^ Murine embryos and hearts were collected in Phosphate Buffered Saline (PBS) on ice at the indicated stages on a stereo microscope (stereo microscope V8, Carl Zeiss, German) and incubated in 10.0% potassium chloride (KCl) (746436, Sigma‐Aldrich, USA) at 4 °C for 5 min to maintain the hearts in diastole. Then it was fixed in 4.0% paraformaldehyde at 4 °C for 6 h. Samples were dehydrated in an ethanol and xylene series and embedded in paraffin. Images were prepared at 3 µm thickness and consecutively collected on slides. HE staining was also performed according to standard procedures as previously described.^[^ 23 ^]^ Images were acquired by slide scanner (Axio Scan Z1, Carl Zeiss, German).

##### Sample Preparation and Micro‐CT Scans

Embryonic hearts were fixed in 4% paraformaldehyde overnight at room temperature before incubation in 25.0% Lugol's solution as a micro‐CT contrast agent overnight at room temperature.^[^ 10 ^]^ Lugol's solution (100%) was prepared from KI (10 g) (746428, Sigma‐Aldrich, USA) plus I_2_ (5 g) (207772, Sigma‐Aldrich, USA) in 100 mL H_2_O and then diluted to 25% with deionized H_2_O, to be approximately isotonic to cardiac tissue. Scanning was performed using a micro‐CT scanner (Xradia 520 Versa, Carl Zeiss, Germany). For each heart, 3600 X‐ray projections were digitized at 0.15° intervals over 180° using 50 kV and 4 W with a 1‐h exposure time. A modified Feldkamp filtered back‐projection algorithm yielding 2.3–3.3 µm *x*‐*y*‐*z* voxels was used to export 990–1050 continuous images.

##### Reconstruction and Visualization

Imaris 9.1.0 (Bitplane, Switzerland, https://www.bitplane.com) software was used to reconstruct exported continuous images into cardiac 3D images and manually mark the ventricular cavity and the great arterial cavity in the 3D image. Imaris 9.1.0 was used to transfer the reconstructed cardiac 3D image into consecutive CT images, which were used in CACCT detection. Imaris 9.1.0 was also used to reconstruct CACCT‐detected cavities and VSD channels.

##### Detection of Cardiac Cavity Regions

The Otsu's method^[^ 24 ^]^ and the region growth algorithm^[^ 25 ^]^ were used to detect cardiac cavity regions. First, the Otsu's method distinguished cardiac tissue from noncardiac tissue (Figures S2 and S3a, Supporting Information). Otsu is an efficient binarization algorithm that can realize the automatic selection of the global threshold through the statistics of the histogram characteristics in a whole image and finally obtain the region of interest from other regions. Then, the cardiac cavity was detected with a region growth algorithm (Figure S3b, Supporting Information). The region growth algorithm can combine pixels with similar properties to obtain cardiac cavity regions as a category. Details are described in the Supporting Information.

##### Construction of 3D Graphical Data Trove

The 3D graphical data trove showed the relationship between hundreds of CT images and the inference principle of all cavities in one heart. The 3D graphical data trove was constructed via five steps: initialization of the seed region, region growth, global searching, and construction of node. 1) When the inner cavity initially appeared, the independent connected area composed of white pixels in the intracavity image was used as the initial seeds. 2) The initial seed was used for region growth to detect updated connected areas on the intracavity of the next frame. 3) The updated connected areas did not include the connected areas that first appeared in the current frame, which was named the independent connected area. Therefore, global searching was needed to detect the independent connected areas. 4) The connected areas obtained from steps (2) and (3) were all the connected areas needed for cavity detection and were used to construct the nodes. These nodes were composed of connected areas and their related information on the current frame. 5) In hundreds of CT images, all nodes constituted a cardiac structural graph that contained the relationship between the connected areas of each frame. Details are described in the Supporting Information.

##### Inference of Cardiac Cavities in Normal Hearts

Ventricular, atrial cavities, and great vessels were automatically detected in all frames and provided the basis for the 3D reconstruction of the anatomical structures, referring to Figures 2b and 4d.

##### Detection of Atrial Cavity Regions

By detecting the atrial cavity region, the corresponding node information of the atrial cavity region could be added to the 3D graphical data trove. The workflow to detect the atrial cavity region (Figure 2b) was as follows: 1) The atrial cavity region was manually marked on the corresponding frame, and then the intracavity region of the frame was divided into two categories: the atrial cavity region and the nonatrial cavity region. 2) According to the node of the corresponding region, the atrial cavity and nonatrial cavity regions were searched on the next frame, and the two types of region obtained from the current frame were input into the discriminant to determine whether atrial cavity and nonatrial cavity regions were connected on the next frame. If there was no connected region, the nodes in the 3D graphical data trove were updated; if there was a connection, the atrial and nonatrial cavity regions were segmented and the edge details were optimized, and then the corresponding nodes in the 3D graphical data trove were updated. Finally, the information of the atrial cavity region was transformed into the corresponding node, and the 3D graphical data trove containing the information of the atrial cavity was obtained. Details are described in the Supporting Information.

##### Detection of Ventricle and Great Vessel Cavities

The corresponding node information of the cavities was added to the 3D graphical data trove following ventricle and great vessel cavity detection, and the connecting relationships of the ventricles and the great vessels were determined. The main steps were as follows: 1) Through mapping of manually marked regions on the corresponding frames, the intracavity regions of the frames were divided into four categories: LV, RV (Figure 4d), aortic, and pulmonary arterial cavity regions. 2) The related region was searched and obtained on the next frame according to the node of the corresponding region, and the attribution of the related region was judged. The corresponding node information was updated in the 3D graphical data trove according to the judgment result. 3) The aorta and pulmonary artery originated from which ventricle was determined according to the relationship between the great vessel nodes and the ventricular cavity nodes in the updated 3D graphical data trove. Details are described in Sections S5.1 and S6 in the Supporting Information.

##### Inference of Ventricular Cavities in Hearts with VSD

Referring to Figure 4d and Sections S5.2 and S5.3 (Supporting Information), by detecting the VSD region, the RV and LV regions could be correctly segmented. The corresponding node information of the VSD, RV, and LV region could be added to the 3D graphical data trove. The main steps were as follows: 1) To accurately detect VSD and segment LV and RV on frames with VSD, a manually marked region was mapped onto the corresponding frame, and then the intracavity region of the frame was divided into two categories: the LV and the RV cavity regions. 2) According to the node of the corresponding region, its related region was searched and obtained on the next frame. A connection between the LV and RV in the related region was then judged. 3) If there was no connection, the attribution of the related region was judged, and the corresponding information of the node in the 3D graphical data trove was updated according to the judgment result; if there was a connection, the LV and RV cavity regions were segmented and the cavity edge details were optimized before the corresponding nodes in the cardiac structure graph could be updated. Finally, information for the ventricular cavity region and VSD was transformed into the corresponding node, and the 3D graphical data trove containing LV, RV and VSD information was obtained. 4) VSD could be detected in the heart according to the updated 3D graphical data trove in steps (2) and (3).

##### Evaluation of Accuracy of CACCT Cavity Detection

The ventricular chamber and related great arteries were detected by CACCT in consecutive CT images data set. The CACCT detected results were manually inspected section by section to mark error regions. There were two types of errors in CACCT segmentation. The first error was characterized as CACCT detected some region that did not belong to target chamber (ground truth) and was named as overflow error. The second error was characterized as CACCT missed part of target chamber (ground truth) and defined as miss error. Overflow and miss regions larger than 15 × 15 µm were manually marked with imaris 9.1.0 section by section. The cavities in interventricular septum and coronary arterial cavities were not regarded as miss regions during manual inspection. Precision and sensitivity were used to evaluate the ability of CACCT as formula (1) and (2)

(1) $$\text{Precision}=\text{TP}\_\text{i}/\left(\text{TP}\_\text{i}+\text{FP}\_\text{i}\right)$$

(2) $$\text{Sensitivity}=\text{TP}\_\text{i}/\left(\text{TP}\_\text{i}+\text{FN}\_\text{i}\right)$$

TP indicates true positive area of cardiac cavities in frame *i*. FP indicates false positive area of cardiac cavities in frame *i*. FN indicates false negative area of cardiac cavities in frame *i*.

##### Statistical Analyses

The data are presented as the means ± SEM and differences between two groups were analyzed by Student's unpaired *t*‐test. A *p* < 0.05 was considered statistically significant.

## Conflict of Interest

The authors declare no conflict of interest.

## Author Contributions

Q.C., Y.N., and S.H. developed pipelines. Q.C., H.J., Z.Y., H.L., L.L., and D.Z. performed animal experiment and acquired micro‐CT data. Q.C. acquired ground truth annotations. S.H., Y.N., Q.C., H.Z., and Q.Y. analyzed results. Q.Y. and L.Z. developed algorithm for cavity tracking. L.Z. developed software for visualization and operation. Y.N., S.H., Q.C., B.Z., and L.Z. wrote the manuscript. Y.N. and S.H. supervised the project.

## Supporting information

Supporting Information

Supplemental Video 1

Supplemental Video 2

Supplemental Video 3

Supplemental Video 4

Supplemental Video 5

Supplemental Video 6

Supplemental Video 7

Supplemental Video 8

Supplemental Video 9
