# Supplementary material for: CACCT: An Automated Tool of Detecting Complicated Cardiac Malformations in Mouse Models
Source: Adv Sci (Weinh). 2020 Feb 20;7(8):1903592. doi: 10.1002/advs.201903592 (PMC7175298; doi:10.1002/advs.201903592)
Supplement: Supplementary file 1 — Supporting Information [file ADVS-7-1903592-s001.pdf]

Copyright WILEY-VCH Verlag GmbH & Co. KGaA, 69469 Weinheim, Germany, 2020.

ADVANCED SCIENCE
Open Access

Supporting Information

for *Adv. Sci.,* DOI: 10.1002/advs.201903592

CACCT: An Automated Tool of Detecting Complicated Cardiac Malformations in Mouse Models

Qing Chu, Haobin Jiang, Libo Zhang, Dekun Zhu, Qianqian Yin, Hao Zhang, Bin Zhou, Wenzhang Zhou, Zhang Yue, Hong Lian, Lihui Liu, Yu Nie, and Shengshou Hu**

CACCT: An automated tool of detecting complicated cardiac malformations in mouse models

Qing Chu[1], Haobin Jiang[1], Libo Zhang[2], Dekun Zhu[1], Qianqian Yin[1], Hao Zhang[3], Bin Zhou[4], Wenzhang Zhou[2], Zhang Yue[5], Hong Lian[1], Lihui Liu[1], Yu Nie[1,*], Shengshou Hu[1,*].

[1]State Key Laboratory of Cardiovascular Disease, Fuwai Hospital, National Center for Cardiovascular Disease, Chinese Academy of Medical Sciences and Peking Union Medical College, Beijing 100037, China.

[2]State Key Laboratory of Computer Science, Institute of Software Chinese Academy of Sciences, Beijing 100089, China.

[3]Heart Center and Shanghai Institution of Pediatric Congenital Heart Diseases, Shanghai Children's Medical Center, National Children's Medical Center, Shanghai Jiao Tong University School of Medicine, Shanghai 200127,China.

[4]State Key Laboratory of Cell Biology, CAS Center for Excellence in Molecular Cell Science, Shanghai Institute of Biochemistry and Cell Biology, Chinese Academy of Sciences (CAS), University of Chinese Academy of Sciences, Shanghai 200031, China.

[5]Department of Cardiovascular Surgery, Union Hospital, Tongji Medical College, Huazhong University of Science and Technology, Wuhan 430022, China.

Correspondence: nieyu@fuwaihospital.org (Y.N.), huss@fuwaihospital.org (S.H.)

Tel: 010-60866139;   Fax: 010-60866092

Tel: 010-88396011;   Fax: 010-88398359

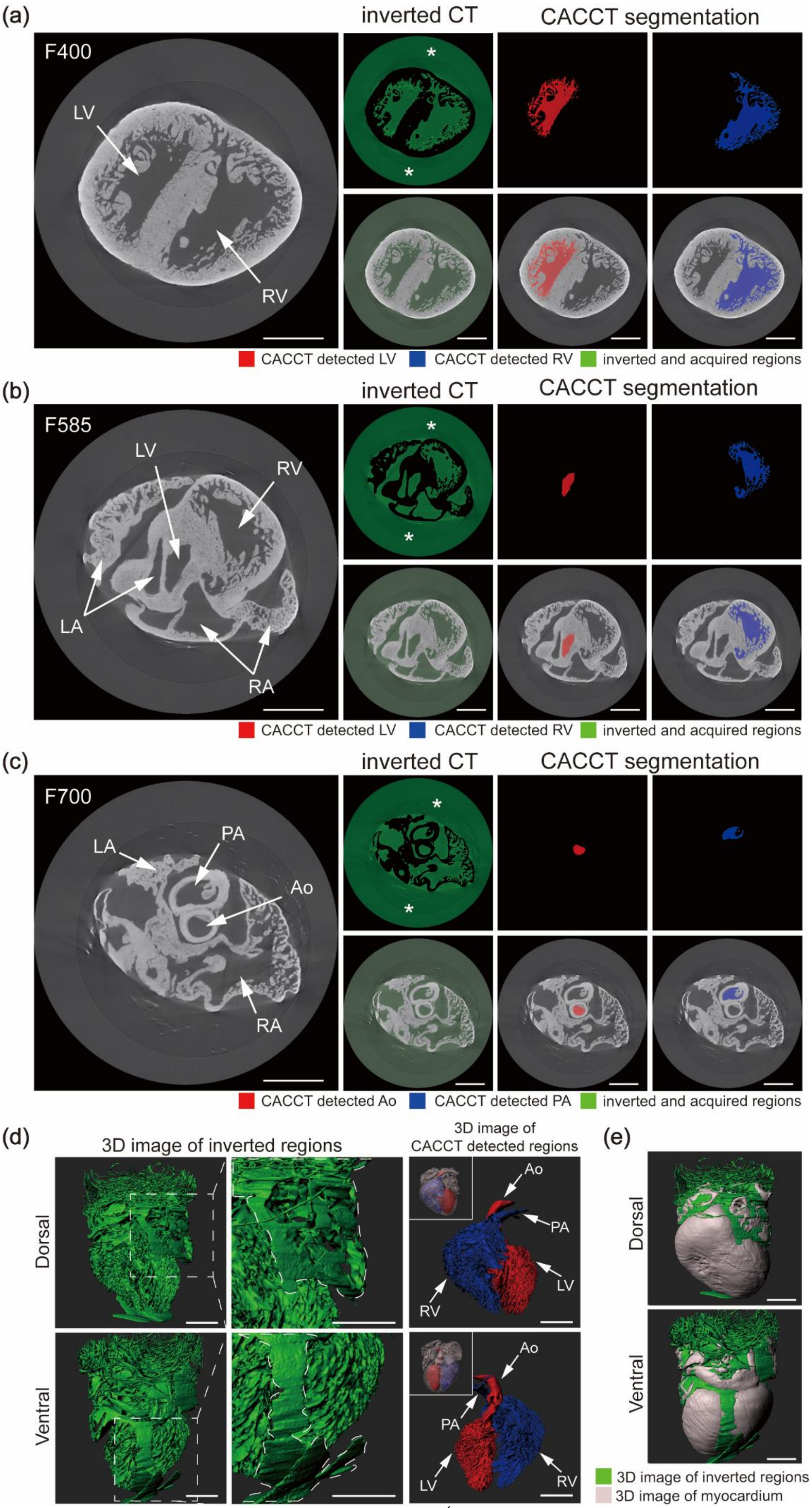

(a)
inverted CT
CACCT segmentation
F400
LV
RV
CACCT detected LV
CACCT detected RV
inverted and acquired regions
(b)
inverted CT
CACCT segmentation
F585
LV
RV
LA
RA
CACCT detected LV
CACCT detected RV
inverted and acquired regions
(c)
inverted CT
CACCT segmentation
F700
LA
PA
Ao
RA
CACCT detected Ao
CACCT detected PA
inverted and acquired regions
(d)
3D image of inverted regions
3D image of CACCT detected regions
Dorsal
Ventral
Ao
PA
LV
RV
(e)
Dorsal
Ventral
3D image of inverted regions
3D image of myocardium

Supplementary Figure 1 Comparison of CACCT segmentation and inversion of CT stacks.

(a) CT transverse section with ventricles showing CACCT segmentation of ventricles and binarization and inversion of CT stacks. The green regions in inverted images were obtained by binarization and inversion, including all cardiac cavities and extracardiac regions. (b) CT transverse section with ventricles and atria showing CACCT segmentation of ventricles and binarization and inversion of CT stacks. (c) CT transverse section with great arteries and atria showing CACCT segmentation of the great arteries and binarization and inversion of CT stacks. (d) 3D ventricular and great arterial cavity images generated by CACCT and 3D cavity images generated by inversion of CT stacks. The insets show 3D myocardium image merged with 3D image of CACCT segmentation. The volume labeled by dotted lines was extracardiac volume detected by in inversion of CT stacks. (e) 3D images of myocardium and inversion of CT stacks. The extracardiac volume covered the surface of 3D image of myocardium, which interfered the tracking of connection of ventricles and the great arteries. F, frame; LV, left ventricle; RV, right ventricle; LA, left atrium; RA, right atrium; Ao, aorta; PA, pulmonary artery; the asterisk indicates extracardiac regions obtained by binarization and inversion.Scale bar: 500 μm.

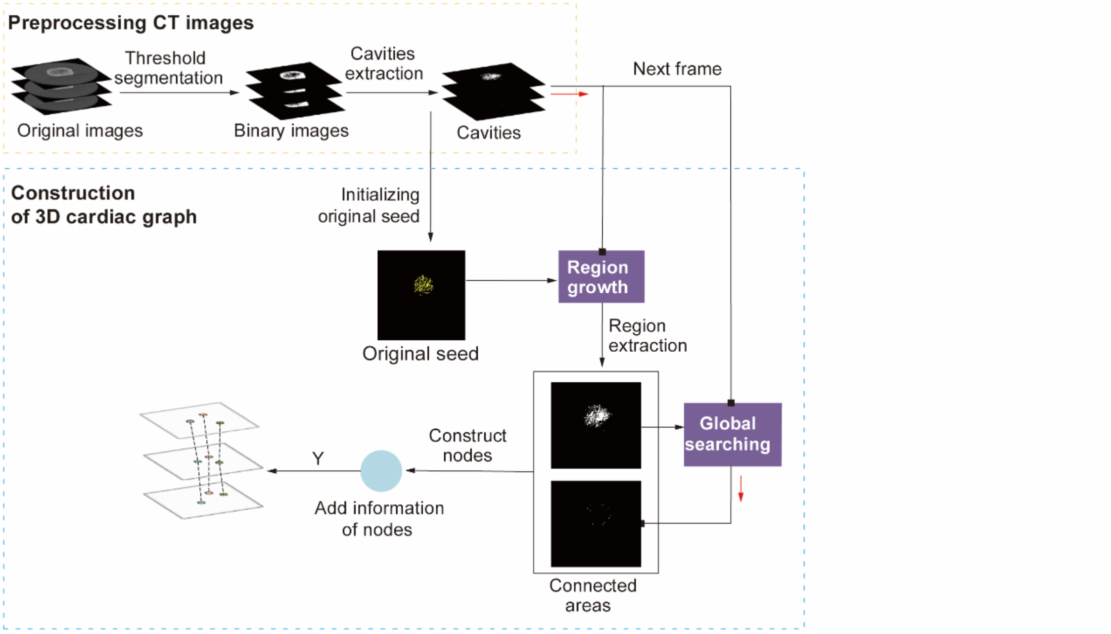

Supplementary Figure 2 Cavity extraction strategy and construction of the 3D graphical data trove. Y, yes.

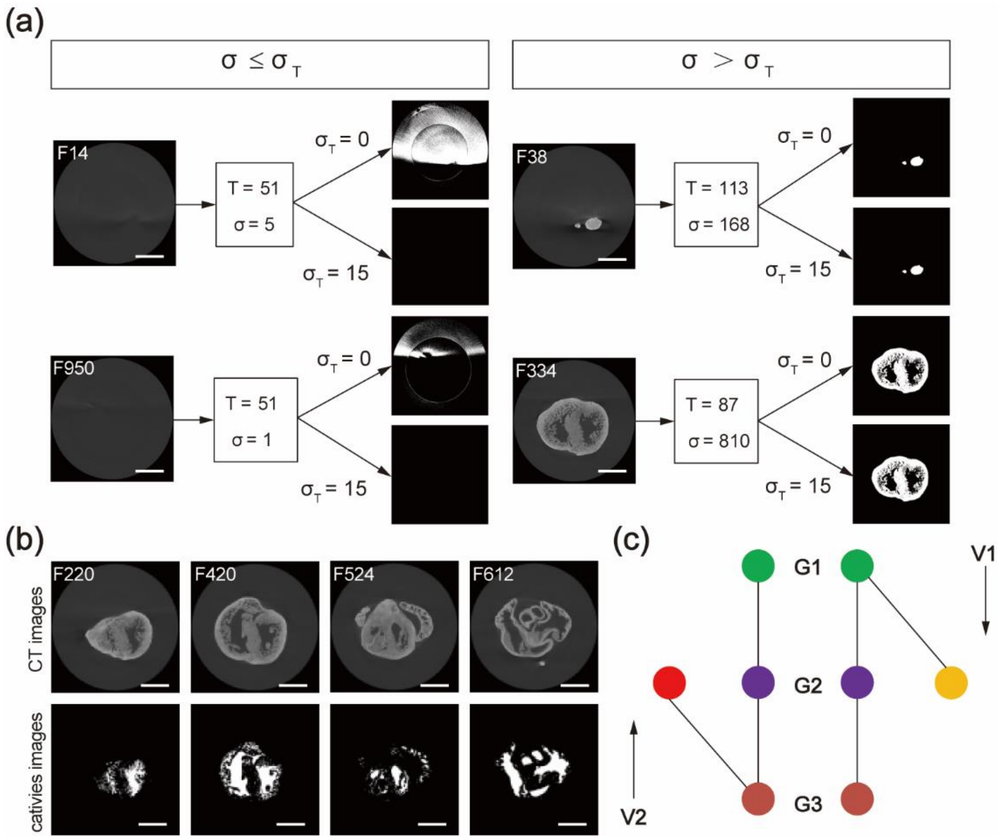

Supplementary Figure 3 CT image pre-processing and original input for CACCT automated cavity detection.

(a) Cardiac tissue detection from CT images of embryonic hearts. σ, the variance in pixels; σ_T, method threshold; F, frame. (b) Extraction of cardiac cavities in CT images. (c) The filtered node optimization procedure. G, frame number of CT images; V, global searching direction. Scale bar: 500 μm.

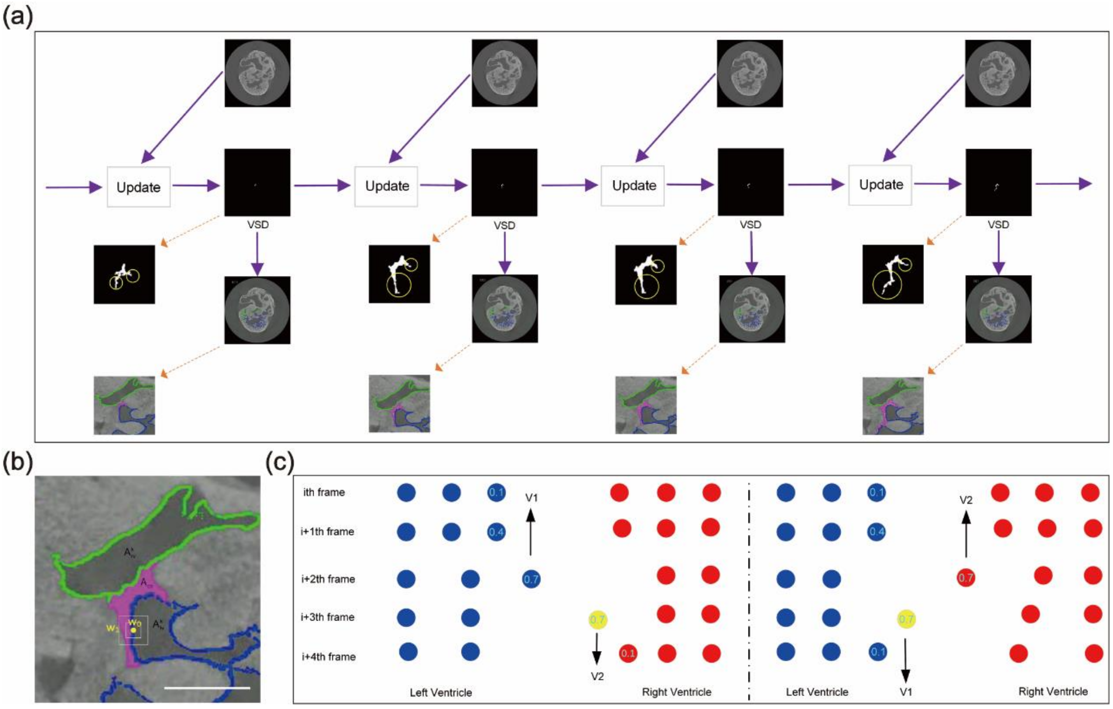

Supplementary Figure 4 CACCT detects VSD regions.

(a) Iteration and optimization of detected VSD regions in consecutive CT images. (b) Schematic showing how the expanded connected areas in A_{cn} are detected and removed. A^{k}_{lv}, connected areas of left ventricle on frame *k*; A^{k}_{rv}, connected areas of right ventricle on frame *k*; A_{cn}, connected areas connecting A^{k}_{lv} with A^{k}_{rv}. (c) Schematic showing oblique VSD detection in consecutive CT images. Circles represent nodes. V, searching direction. Scale bar: 200 μm.

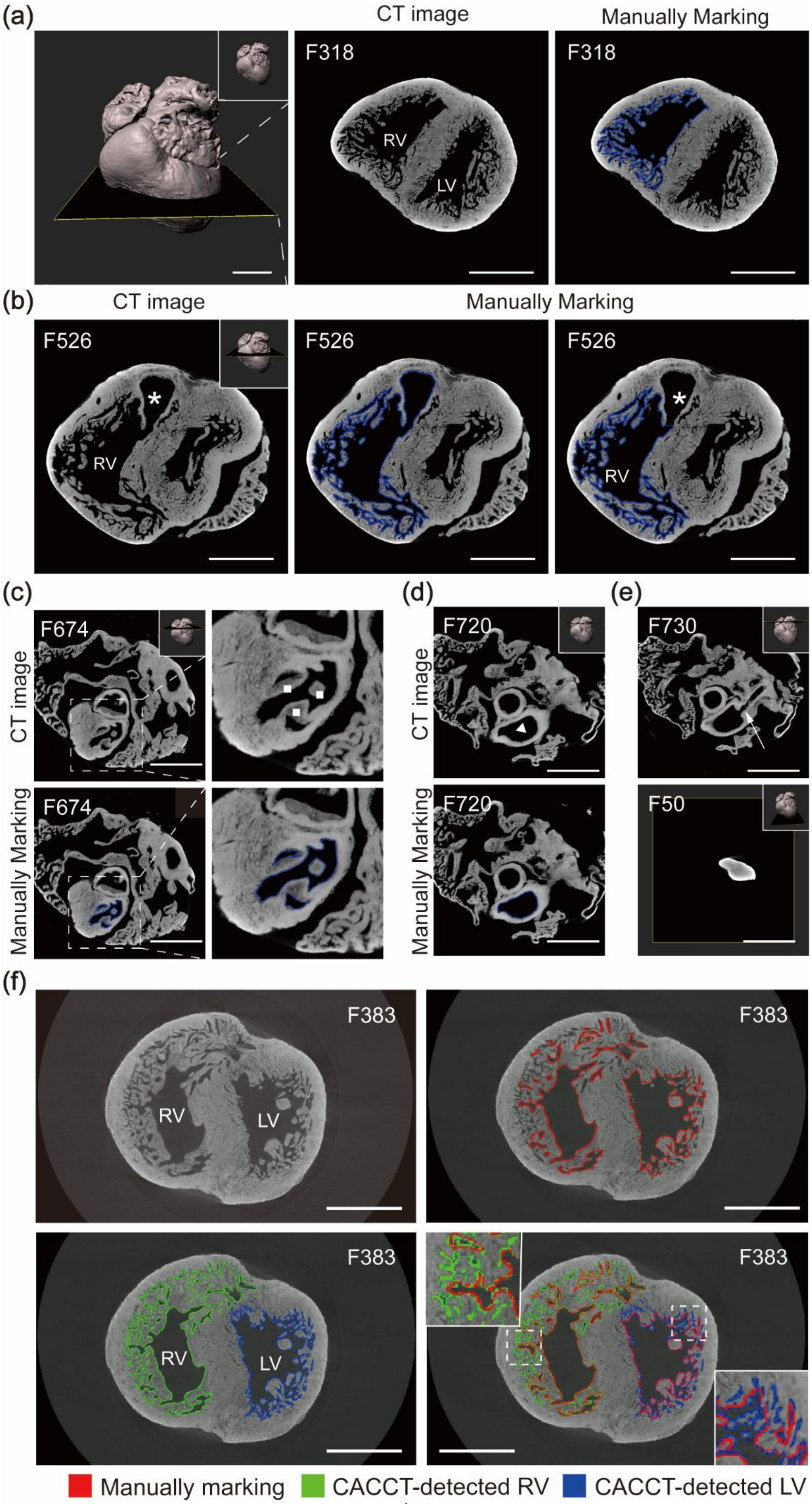
(a)
CT image
Manually Marking
F318
RV
LV
(b)
CT image
Manually Marking
F526
*
RV
(c)
CT image
Manually Marking
F674
(d)
F720
(e)
F730
F50
(f)
F383
RV
LV
Manually marking
CACCT-detected RV
CACCT-detected LV

Supplementary Figure 5 Manual marking of ventricular and the great arterial cavities in consecutive CT images.

(a) Transverse section of the CT image dataset showing manually marked ventricles (blue lines). The inset shows a 3D myocardium image generated by reconstruction of a consecutive CT image dataset without image processing. F, frame. (b) Manually marked right ventricular cavities without right atrial cavities (asterisk). The inset shows the location of the marked transverse section. (c) Manually marked great arterial cavities connected to the marked right ventricle on sections with semilunar valves (squares). The inset shows the location of the marked transverse section. (d) A manually marked great arterial cavity on sections without semilunar valves. (e) Transverse sections before and after manual marking. The inset shows the location of the marked transverse section, the triangle indicates the pulmonary artery; the arrow indicates the bifurcation of the pulmonary artery. (f) Transverse section of the cardiac CT image dataset showing manually marked (red lines) and CACCT detected (green: right ventricle, blue: left ventricle) ventricular cavities. RV, right ventricle; LV, left ventricle; F, frame. Scale bar: 500 μm.

Supplementary methods

1. Manually marking ventricular cavities and the great vessels

The ventricular cavity and the related great arterial cavity were used to represent the corresponding outflow tract (OFT). First, a new surface module was created, and transverse section was selected as the annotation image. After observing all images of the heart, an image with only two specific ventricles was selected as the initial image of ventricular cavities (Supplementary Figure 5a). Then, the area of the two ventricular cavities on this image was determined. A ventricular cavity was randomly selected on the initial image and the inner and outer contours of all areas of the ventricular cavity were marked with a "magic wand" with 10.0% reductivity and 10.0% fault tolerance (Supplementary Figure 5a). Then, the inner and outer edges of all areas of the ventricular cavity were marked on the initial image with the "magic wand", image by image toward the bottom of the heart. As the images moved toward the bottom of the heart, an image was found on which the ventricle and the atria connected (Supplementary Figure 5b). The "magic wand" would not only recognize the contour of the ventricular cavity but would also recognize the contour of the atrial cavity connected with the ventricular cavity. The marked contour of the atrial cavity was deleted on these images to avoid the interference of atria in the observation of the OFT (Supplementary Figure 5b). As the image moved toward the bottom of the heart, the ventricle evolved into the great artery. The inner and outer contour of all areas of the great arterial cavity, arising from the ventricular cavity, were marked with a "magic wand" (Supplementary Figure 5c,d). Marking was stopped on the image where the great artery bifurcates (Supplementary Figure 5e). Then, the inner and outer edges of all areas of the ventricular cavity were with a "magic

wand" on the initial image of ventricular cavities, image by image toward the apex until the area of the ventricular cavity disappeared on the image (Supplementary Figure 5e).

2. Detection of cardiac cavity regions

For a computed tomography (CT) image, first, the black border area around the image was removed by the threshold segmentation method (pixel gray-value=255), and then the remaining areas, expressed as A_n, were extracted using the OTSU method to obtain a binary image (the black part was the area of cardiac cavities and external region of the heart, and the white part was the cardiac tissue) (Supplementary Figure 3a). Before the appearance of the ventricular cavity (observed from the apex to the bottom of the heart), the differences between regional pixels on images were not obvious, and the variance, expressed as σ, was also small. As segmentation error occurred by using the OTSU method, a method threshold σ_T was used. If $\sigma > \sigma_T$, A_n would be threshold segmented; otherwise, A_n would be set to black (Supplementary Figure 3a). The black pixels were selected as seeds in A_n. Based on the region growth algorithm, all the connected areas of the black regions expressed as A^c were obtained. The maximum connected areas were removed from A^c, and the remaining connected areas represented the cardiac cavity region remembered as A_{lu} (Supplementary Figure 3b). Cardiac cavity images were obtained by iterating all images of the cardiac CT images with the above methods.

3. Construction of a three-dimensional (3D) graphical data trove

A connected area was defined as a node, and each node contained all types of information of

the connected area, including the number of the frame, pixel vector (internal pixel vector, edge pixel vector), area size, and center coordinates.

It was assumed that connected areas of the cardiac cavities region $\{A_j^i | j = 1,2,..,N^i\}$ were obtained on frame *i* and A_j^i meant the connected area of node N_i on frame *i*, and N_i represented the number of these connected areas. These connected areas were used as seeds $\{S_j^{i+1} = A_j^i | j = 1,2,...,N^i\}$ for the growth of the corresponding connected areas on frame *i+1*. If the connected areas $\{A_j^{i+1} | j = 1,2,...,N_k^{i+1}\}$ on frame *i+1* were obtained based on the seed *k* on frame *i*, the connected areas were the son node of node *k*, and node *k* on frame *i* was the parent node of the N_k^{i+1} node on frame *i+1*.

Moreover, the global search method might obtain independent connected areas $\{A_j^{i+1} | j = 1,2,...,D^{i+1}\}$ from the cardiac cavity region on frame *i+1*, which had no parent nodes on frame *i*. The independent connected areas and the connected areas obtained by seeds $\{S_j^{i+1} = A_j^i \mid j = 1,2,..., N^i\}$ constituted all nodes on frame *i+1* (Supplementary Figure 3c).

Then, the nodes obtained on frame *i+1* were used as seeds of frame *i+2*, and all nodes on frame *i+2* were obtained as mentioned above. The 3D graphical data trove, Ω_G, was constructed by iterating all frames of the cardiac CT images of one heart. In addition, all the original connected areas obtained were based on the cardiac cavity region as seeds, without parent nodes.

For detecting the targeted part of intracardiac regions, it was necessary to determine which targeted node belonged to each part of the heart (attribution of the node). Therefore, 10 attributing markers (M) were defined for each node (Supplementary Table 1).

4. Detection of the atrial cavity region

The left and right atrium areas were assumed to be A_u^{kl} and A_u^{kr} on frame *k*, respectively, and were also called atrium areas and denoted as V. The remaining regions of the cardiac cavity region on the images were taken as non-atrium areas and denoted as B. The initial areas of A_u^{kl} and A_u^{kr} were obtained by mapping the manual annotations onto the corresponding image and binary image. Before detecting the atrial cavity, the nodes of small areas were filtered out to avoid the interference of small areas, and these filtered nodes were optimized after marking the atrial and ventricular cavity and great arterial cavity (Supplementary methods Optimization of filtered nodes).

During atrial cavity region detection, the similarity between the node on the latter frame with the atrium regions and non-atrium regions on the previous frame was assessed to determine the attribution of the node on the latter frame. This similarity formula was defined as follows:

$$\begin{cases} r_v = \dfrac{s^v}{N(A_{p,v})} \\ r_b = \dfrac{s^b}{N(A_{p,b})} \end{cases} \qquad (1)$$

r_v is the similarity between the node and atrium regions of the previous frame (frame *k*), r_b is the similarity between the node and non-atrium regions of the previous frame (frame *k*); $s^v s^v$ and s^b represent the overlapped area between the connected area and atrium regions and non-atrium regions of the previous frame (frame *k*), respectively; $A_{p,v}$ and $A_{p,b}$ represent the part of the parent node of the current node overlapped with atrium region and non-atrium region part, respectively; and $N(\bullet)$ represents the area of the corresponding connected area.

Connections between the atrial and ventricular cavities or other cardiac cavity regions

meant that the connected area of the atrial cavity consisted of both atrial and non-atrial regions. When atrium regions were updated on the latter frame, the attribution of all the nodes on the latter frame were determined as follows:

$$M_t^{k+1}=\begin{cases}2,r_{v,t}^{k+1}>\sigma_v,r_{b,t}^{k+1}<\sigma_b\\ 7,r_{v,t}^{k+1}>0\\ 0,others\end{cases} \quad (2)$$

M_t^{k+1} represents the attribution of node *t* on frame *k+1*, M_t^{k+1} = 2 indicates that the node belongs to the atrial regions, M_t^{k+1} = 7 indicates that the node is composed of part of the atrial and non-atrial regions, and 0 indicates that the node belongs to the non-atrial regions. σ_v and σ_b represent similarity thresholds compared with atrial and non-atrial regions, respectively.

The connected area $A_{it,t}^{k+1}$ represented by node *t* on frame *k+1* was assumed to contain both the atrial and non-atrial regions, and that M_t^{k+1} = 7; thus it was necessary to distinguish and segment the atrial and non-atrial areas in the connected areas and update the atrial V^{k+1}, the non-atrial B^{k+1} and the set of nodes $\{G_j^{k+1}|j=1,2,...,N_g^{k+1}\}$.

$$\begin{cases}A_{it,t}^{k+1}=A_t^{k+1}\cap V^k\\ C_t^{k+1}=A_t^{k+1}-A_{it,t}^{k+1}\end{cases} \quad (3)$$

$A_{it,t}^{k+1}$ represents the intersection of the connected area A_t^{k+1} of node *t* on frame *k+1* with the atrial connected areas V^k on frame *k*; C_t^{k+1} represents the remaining part of A_t^{k+1} after $A_{it,t}^{k+1}$ was removed.

New connected areas $\Omega_A^{k+1,t}=\{A_1^{new},...,A_m^{new},...A_n^{new}\}$ (n = $N_{new,t}^{k+1}$) were obtained by the region growth algorithm in C_t^{k+1} and $\forall A_m^{new}\in\Omega_A^{k+1,t}$:

$$A_{new,t}^{k+1} = \begin{cases} A_{new,t}^{k+1} \cup A_m^{new}, & A_m^{new} \cap B^k \neq O \\ A_{new,t}^{k+1}, \text{others} \end{cases} \quad (4)$$

$A_{new,t}^{k+1}$ represents the remaining part after the atrial connected areas on frame *k* were removed, and $A_{new,t}^{k+1}$ is initially empty. B^k represents the background area of the atrial cavity region on frame *k*.

The previous content of the node C_t^{k+1} was deleted, the region-related information of $A_{new,t}^{k+1}$ was recorded, and then the connection relationship between parent and son nodes was updated according to whether there was intersection of parent with son nodes. The atrial and non-atrial regions on frame *k+1* were obtained by judging the atrial and non-atrial attribution of all nodes on frame *k+1*.

$$V^{k+1} = \begin{cases} V^{k+1}, M_t^{k+1} = 0 \\ V^{k+1} \cup A_{new,t}^{k+1}, M_t^{k+1} = 7 \\ V^{k+1} \cup A_t^{k+1}, M_t^{k+1} = 2 \end{cases} \quad (5)$$

$$B^{k+1} = A^{k+1} - V^{k+1} \quad (6)$$

V^{k+1} represents the atrial connected areas on frame *k+1*. $A_{new,t}^{k+1}$ represents the remaining part after the atrial connected areas on frame *k* were removed. A_t^{k+1} represents node *t* on frame *k*. M_t^{k+1} represents the attribution judgment of node *t* on frame *k*, M_t^{k+1} = 2 indicates that the node belongs to the atrial region, M_t^{k+1} = 7 indicates that the connected area of the node is composed of atrial and non-atrial regions, and M_t^{k+1} = 0 indicates that the node belongs to the non-atrial region.

Atrial cavity region detecting algorithm:

Input：the atrial region V_l and the number F_l of the left atrial original image, the atrial region V_r and the number F_r of the right atrial original image, the 3D graphical data trove Ω_g, the set of original CT images Is, the amount of original CT images N and the set of the cardiac cavities images Ip.

The number of the first atrial image F0 = min (Fl, Fr), atrial region $V^{F0} = 0$, nonatrial region $B^{F0} = 0$

For iter = F0: N

$$V^{iter} = \begin{cases} V^{iter} \bigcup V^{l}, iter = F_l \\ V^{iter} \bigcup V^{r}, iter = F_r \end{cases}$$

If iter = F_1 or iter = F_r, according to set of nodes on frame *iter* expressed as Ω_g^{iter} and V^{iter}, obtained B^{iter}.

Obtaining the number of nodes $N_g^{iter} = len(\Omega_g^{iter})$ in the 3D graphical data trove Ω_g, on frame *iter*

For *j* = 0 to N_g^{iter}

When calculating the similarity r_v between the connected area of the node G_j^{iter} and the atrial region V^{iter}, and the similarity r_b between G_j^{iter} and the non-atrial region B^{iter} an attribution marker of the node M_j^{iter}, was obtained.

If M_j^{iter} = 7, obtain $A_{new,j}^{iter}$

According to formula (5) and (6), update V^{iter+1}, B^{iter+1} and G_j^{iter}; if M_j^{iter} = 7, then M_j^{iter} = 0

End

End

Output: updated 3D graphical data trove Ω_g, attributing marker M

5. Detection of ventricular cavity region

5.1 Detection of normal ventricular cavity

For detecting the ventricular cavity region, the LV cavity and RV cavity regions on frame *k* were assumed to be A_{lv}^{k} and A_{rv}^{k}, and the corresponding set of nodes were $\Omega_{g,lv}^{k}$ and $\Omega_{g,rv}^{k}$.

A similarity formula was defined to determine the attribution of all son nodes in the latter frame or all parent nodes on the previous frame, assuming when $\Omega_{nt} = \Omega_{g}^{k-1}$ or $\Omega_{nt} = \Omega_{g}^{k+1}$, $\forall G \in \Omega_{nt}$:

$$\begin{cases} A_{it,lv} = A_{G} \cap A_{lv}^{k} \\ A_{it,rv} = A_{G} \cap A_{rv}^{k} \\ d_1 = \dfrac{N(A_{it,lv})}{N(A_{lv}^{k})} \\ d_2 = \dfrac{N(A_{it,rv})}{N(A_{rv}^{k})} \end{cases} \tag{7}$$

A_G is the connected area of node *G*. $A_{it,lv}$ and $A_{it,rv}$ represent the intersection of the corresponding connected area of node *G* with the left ventricular (LV) cavity A_{lv}^{k} and right ventricular (RV) cavity A_{rv}^{k}, respectively, on the parent (or son) frame of node *G*. d_1 and d_2 represent the similarity between node *G* with the LV cavity region and RV cavity region, respectively; $N(\bullet)$ represents the area of the corresponding connected area.

All son nodes or parent nodes of the 3D graphical data trove without congenital heart defects were directly transmitted: the son or parent nodes obtained from the LV still belonged to the LV region, and the son or parent nodes obtained from the RV still belonged to the RV.

Therefore, the principle of the attribution of nodes is given:

$$M_G = \begin{cases} 3, d_1 > d_2 \ \& \ d_1 > 0 \\ \\ 4, d_2 > d_1 \ \& \ d_2 > 0 \end{cases} \quad (8)$$

M_G indicates the attribution of node *G*; M_G = 3 indicates that node G belongs to the LV cavity region; M_G = 4 indicates that node *G* belongs to the RV cavity region.

First, the initial ventricular cavity regions were obtained by mapping the manually marked initial ventricular cavity image on the corresponding image and the binary image. Then, the next ventricular cavity region on the son (or parent) frame was detected based on the initial ventricular cavity region. The iteration process was repeated to detect the whole ventricular cavity region and the 3D graphical data trove of one ventricular cavity was updated.

Ventricular cavity detection algorithm:

Input: the manually marked initial LV cavity image A_{lv}^{k0}, the manually marked initial RV cavity image A_{rv}^{k0}, the number of the original image *K0*, the 3D graphical data trove Ω_g, the set of original CT images Is, the number of original CT images N, the set of the cardiac cavities images Ip, the set of LV nodes $\Omega_{all, lv}$ and the connected areas $A_{all, lv}$, the set of RV nodes$\Omega_{all, rv}$ and the connected areas $A_{all, rv}$.

---

1) The manually marked initial LV cavity image A_{lv}^{k0} and the manually marked initial RV cavity image A_{rv}^{k0}, obtain nodes $\Omega_{g,lv}^{k0}$ and $\Omega_{g,rv}^{k0}$, whose attributing marker $M^{k0} = 0$.

$A_{all,lv} \leftarrow A_{all,lv} + A_{lv}^{k0}$, $A_{all,rv} \leftarrow A_{all,rv} + A_{rv}^{k0}$, $\Omega_{all,lv} \leftarrow \Omega_{all,lv} + \Omega_{lv}^{k0}$, $\Omega_{all,rv} \leftarrow \Omega_{all,rv} + \Omega_{rv}^{k0}$

$A_{lv} \leftarrow A_{lv}^{k0}$, $A_{rv} \leftarrow A_{rv}^{k0}$, $\Omega_{lv} \leftarrow \Omega_{lv}^{k0}$, $\Omega_{rv} \leftarrow \Omega_{rv}^{k0}$

2) Search forward from frame *k0*.

For $k = k0$ to 1

$A_{lv}^{k} \leftarrow A_{lv}$, $A_{rv}^{k} \leftarrow A_{rv}$, $\Omega_{g,lv}^{k} \leftarrow \Omega_{g,lv}$, $\Omega_{g,rv}^{k} \leftarrow \Omega_{g,rv}$

$A_{lv} = O$, $A_{rv} = O$, $\Omega_{g,lv} = O$, $\Omega_{g,rv} = O$

According to the 3D graphical data trove Ω_g, obtain parent nodes $\Omega_{g,parent}^{k-1}$, whose attributing marker M = 0, of $\Omega_{g,lv}^{k}$ and $\Omega_{g,rv}^{k}$. The amount of the $\Omega_{g,parent}^{k-1}$ is $N_{g,parent}^{k-1}$.

If $N_{g,parent}^{k-1} = 0$

End the circulation.

End

For $j = 0$ to $N_{g,parent}^{k-1}$

$G = G_j$, $G_j \in \Omega_{g,parent}^{k-1}$, the connected areas of node G are A_G

According to formula (7) and (8), judge the attribution of nodes:

If $M_2 = 3$, $A_{lv} \leftarrow A_{lv} \cup A_G$, $\Omega_{g,lv} \leftarrow \Omega_{g,lv} + G$

If $M_2 = 4$, $A_{rv} \leftarrow A_{rv} \cup A_G$, $\Omega_{g,rv} \leftarrow \Omega_{g,rv} + G$

End

$A_{all,lv} \leftarrow A_{all,lv} + A_{lv}$, $A_{all,rv} \leftarrow A_{all,rv} + A_{rv}$,

$\Omega_{all, lv} \leftarrow \Omega_{all, lv} + \Omega_{g, lv}$, $\Omega_{all, rv} \leftarrow \Omega_{all, rv} + \Omega_{g, rv}$

End

3) Search backward from frame *k0*.

$A_{lv} \leftarrow A^{k0}_{lv}$, $A_{rv} \leftarrow A^{k0}_{rv}$, $\Omega_{g, lv} \leftarrow \Omega^{k0}_{g, lv}$, $\Omega_{g, rv} \leftarrow \Omega^{k0}_{g, rv}$

For k = *k0* to *N*

$A^{k}_{lv} \leftarrow A_{lv}$, $A^{k}_{rv} \leftarrow A_{rv}$, $\Omega^{k}_{g, lv} \leftarrow \Omega_{g, lv}$, $\Omega^{k}_{g, rv} \leftarrow \Omega_{g, rv}$

$A_{lv} = O$, $A_{rv} = O$, $\Omega_{g, lv} = O$, $\Omega_{g, rv} = O$

According to the 3D graphical data trove Ω_g, obtain son nodes $\Omega^{k+1}_{g, son}$, whose attributing marker M = 0, of $\Omega^{k}_{g, lv}$ and$\Omega^{k}_{g, rv}$. The amount of the $\Omega^{k+1}_{g, son}$ is $N^{k+1}_{g, son}$.

If $N^{k+1}_{g, son} = 0$

End the circulation.

End

For *j* = 0 to $N^{k+1}_{g, son}$

$G = G_j$, $G_j \in \Omega^{k+1}_{g, son}$, the connected areas of node G are A_G

According to formula (7) and (8), judge the attribution of nodes:

If $M_2 = 3$, $A_{lv} \leftarrow A_{lv} \cup A_G$, $\Omega_{g, lv} \leftarrow \Omega_{g, lv} + G$

If $M_2 = 4$, $A_{rv} \leftarrow A_{rv} \cup A_G$, $\Omega_{g, rv} \leftarrow \Omega_{g, rv} + G$

End

$A_{all, lv} \leftarrow A_{all, lv} + A_{lv}$, $A_{all, rv} \leftarrow A_{all, rv} + A_{rv}$,

$\Omega_{all, lv} \leftarrow \Omega_{all, lv} + \Omega_{g, lv}$, $\Omega_{all, rv} \leftarrow \Omega_{all, rv} + \Omega_{g, rv}$

End

Output: the set of LV cavity nodes $\Omega_{all, lv}$ and the connected areas $A_{all, lv}$, the set of RV cavity nodes $\Omega_{all, rv}$ and the connected areas $A_{all, rv}$, attributing marker M.

5.2 Detection of ventricular cavity on images with paralleled VSD

For all son nodes or parent nodes with VSD, there were three connecting situations of ventricular cavities: (i) direct transmission, where the son or parent nodes obtained from the LV still belonged to the LV region, and the son or parent nodes obtained from the RV (or LV) still belong to the RV (or LV); (ii) directly connected, where the son node or parent nodes obtained from the LV and RV are directly connected, which means part of these nodes belonged to RV, while the rest belonged to LV; (iii) the son node or parent nodes obtained from LV or RV connected to nonventricular cardiac cavities regions.

Therefore, the updated principle of the attribution of nodes is given to determine whether the LV and the RV are directly connected:

$$M_G = \begin{cases} 8, d_1 > vR0 \& d_2 > vR1 \\ 3, d_1 > d_2 \& d_1 > 0 \\ 4, d_2 > d_1 \& d_2 > 0 \end{cases} \tag{9}$$

M_G represents the attribution of node *G*. M_G = 8 indicates that the son node or parent node *G* is not only from a node in LV but also from a node in RV on the current image. M_G = 8 also indicates that the LV and the RV are directly connected in the frame *k-1* or *k+1*. M_G = 3 indicates that node *G* belongs to the LV region. M_G = 4 indicates that node *G* belongs to the RV region. vR0 and vR1 represent the similarity thresholds between node *G* and the LV and RV, respectively.

When M_G = 8, the LV and the RV were directly connected in frame *k-1* or *k+1*. In this case, it was necessary to distinguish and segment LV and RV. First, the connected area A_G corresponding to node *G* was acquired. According to A^k_{lv} and A^k_{rv}, C_G, the additional area in A_G compared to LV and RV was obtained:

$$\begin{cases} A_{it} = A_G \cap (A^k_{lv} \cup A^k_{rv}) \\ C_G = A_G - A_{it} \end{cases} \tag{10}$$

A_{it} represents the intersection of A_G with A^k_{lv} and A^k_{rv}. The intersection was removed from A_G and the remaining areas were C_G. All connected areas $\{A^G_{new,1}, A^G_{new,2}, A^G_{new,3}, ...\}$ were detected from C_G, and all connected areas A_{cn}, connecting A^k_{lv} with A^k_{rv}, were found in C_G (Supplementary Figure 4a).

Some connected areas in A_{cn} originated from the expansion of edges of A^k_{lv} and A^k_{rv}. If the expanded connected areas in A_{cn} were not removed, after *m* evolving steps, A^{k+m}_{cn} (or A^{k-m}_{cn}) would erode some connected areas belonging to A^{k+m}_{lv} and A^{k+m}_{rv} (or "A^{k-m}_{lv} and A^{k-m}_{rv}").

The edge $E_{cn, lv}$ belonged to both A_{cn} and A^k_{lv}, edge $E_{cn,rv}$ belonged to A_{cn} and A^k_{rv}, and the other edge $E_{cn, uc}$ belonged to A_{cn}, which did not adhere to any connected areas of ventricular cavities (Supplementary Figure 4b). Each point on edge $E_{cn, lv}$ and $E_{cn, rv}$ was judged, regardless of whether the point on edge $E_{cn, uc}$ existed in the window w_1. If the condition was satisfied, the point on $E_{cn, lv}$ or $E_{cn, rv}$ would be attributed to the corresponding ventricular cavities and was expanding to A_{cn}, and the area of window w_0, whose center was the point on edge $E_{cn, lv}$ and $E_{cn, rv}$, would be fused into the corresponding ventricular region. If the condition was not satisfied, the point on $E_{cn, lv}$ or $E_{cn, rv}$ would be attributed to A_{cn} (Supplementary Figure 4b).

By removing the connected area expanded from A_{lv}^{k} or A_{rv}^{k} in A_{cn}, the final connected area $A_{fl,\ cn}$ was obtained, which connected A_{lv}^{k} with A_{rv}^{k}. After removing $A_{fl,\ cn}$, several connected areas were detected from the remaining area, expressed as B_G. The remaining connected areas were attributed to the corresponding ventricular cavity on frame *k+1* or *k-1* according to the similarity between the connected area with LV (or RV) (see formula (7)).

Ventricular cavity region detecting algorithm (with paralleled VSD):

Input: the original image of the LV cavity region A_{lv}^{k0}, the original image of the RV cavity region A_{rv}^{k0}, the number of the original image K0, the 3D graphical data trove Ω_g, the set of original CT images Is, the number of original CT images N, the set of the cardiac cavity images Ip, the set of LV nodes $\Omega_{all,\ lv}$ and connected areas $A_{all,\ lv}$, the set of RV nodes $\Omega_{all,\ rv}$ and the connected areas $A_{all,\ rv}$.

---

1) The original image of the LV cavity region A_{lv}^{k0} and the original image of the RV cavity region A_{rv}^{k0}, obtain nodes $\Omega_{g,\ lv}^{k0}$ and$\Omega_{g,\ rv}^{k0}$, whose attributing marker $M^{k0} = 0$.

   $A_{all,\ lv} \leftarrow A_{all,\ lv} + A_{lv}^{k0}$, $A_{all,\ rv} \leftarrow A_{all,\ rv} + A_{rv}^{k0}$

   $\Omega_{all,\ lv} \leftarrow \Omega_{all,\ lv} + \Omega_{g,\ lv}^{k0}$, $\Omega_{all,\ rv} \leftarrow \Omega_{all,\ rv} + \Omega_{g,\ rv}^{k0}$

   $A_{lv} \leftarrow A_{lv}^{k0}$, $A_{rv} \leftarrow A_{rv}^{k0}$, $\Omega_{g,\ lv} \leftarrow \Omega_{g,\ lv}^{k0}$, $\Omega_{g,\ rv} \leftarrow \Omega_{g,\ rv}^{k0}$

2) Search forward from frame *k0*.

For k = *k0* to 1

   $A_{lv}^{k} \leftarrow A_{lv}$, $A_{rv}^{k} \leftarrow A_{rv}$, $\Omega_{g,\ lv}^{k} \leftarrow \Omega_{g,\ lv}$, $\Omega_{g,\ rv}^{k} \leftarrow \Omega_{g,\ rv}$

   $A_{lv} = O$, $A_{rv} = O$, $\Omega_{g,\ lv} = O$, $\Omega_{g,\ rv} = O$

According to the data structure Ω_g, obtain parent nodes $\Omega_{g,\ parent}^{k-1}$, whose attributing

---

marker M = 0, of $\Omega^{k}_{g,\,lv}$ and $\Omega^{k}_{g,\,rv}$. The amount of the $\Omega^{k-1}_{g,\,parent}$ is $N^{k-1}_{g,\,parent}$.

If $N^{k-1}_{g,\,parent} = 0$

End the circulation.

End

For $j = 0$ to $N^{k-1}_{g,\,parent}$

$G = G_j$, $G_j \in \Omega^{k-1}_{g,\,parent}$, the connected areas of node G are A_G

According to formula (7) and (9), judge the attribution of nodes:

If $M_2 = 3$, $A_{lv} \leftarrow A_{lv} \cup A_G$, $\Omega_{g,\,lv} \leftarrow \Omega_{g,\,lv} + G$

If $M_2 = 4$, $A_{rv} \leftarrow A_{rv} \cup A_G$, $\Omega_{g,\,rv} \leftarrow \Omega_{g,\,rv} + G$

If $M_G = 8$, detect area $A_{fl,\,cn}$, which connects A^{k}_{lv} and A^{k}_{rv}. Removing $A_{fl,\,cn}$ from A_G, obtaining all connected areas $\{A_{re,\,1}, A_{re,\,2}, \ldots\}$ from the remaining area and $VA_{re,\,h} \in \{A_{re,\,1}, A_{re,\,2}, \ldots\}$. Calculating similarity d_1 between $A_{re,\,h}$ and A^{k}_{lv} and calculating similarity d_2 between $A_{re,\,h}$ and A^{k}_{rv}. Obtaining the attributing marker of $A_{re,\,h}$, M_2.

If $M_2 = 1$, $A_{lv} \leftarrow A_{lv} \cup A_{re,\,h}$, $\Omega_{g,\,lv} \leftarrow \Omega_{g,\,lv} + G$

If $M_2 = 0$, $A_{rv} \leftarrow A_{rv} \cup A_{re,\,h}$, $\Omega_{g,\,rv} \leftarrow \Omega_{g,\,rv} + G$

End

$A_{all,\,lv} \leftarrow A_{all,\,lv} + A_{lv}$, $A_{all,\,rv} \leftarrow A_{all,\,rv} + A_{rv}$,

$\Omega_{all,\,lv} \leftarrow \Omega_{all,\,lv} + \Omega_{g,\,lv}$, $\Omega_{all,\,rv} \leftarrow \Omega_{all,\,rv} + \Omega_{g,\,rv}$

End

3) Search backward from frame *k0*.

$A_{lv} \leftarrow A^{k0}_{lv}$, $A_{rv} \leftarrow A^{k0}_{rv}$, $\Omega_{g,\,lv} \leftarrow \Omega^{k0}_{g,\,lv}$, $\Omega_{g,\,rv} \leftarrow \Omega^{k0}_{g,\,rv}$

For k = *k0* to *N*

$A^k_{lv} \leftarrow A_{lv}$, $A^k_{rv} \leftarrow A_{rv}$, $\Omega^k_{g, lv} \leftarrow \Omega_{g, lv}$, $\Omega^k_{g, rv} \leftarrow \Omega_{g, rv}$

$A_{lv} = O$, $A_{rv} = O$, $\Omega_{g, lv} = O$, $\Omega_{g, rv} = O$

According to the 3D graphical data trove Ω_g, obtain son nodes $\Omega^{k+1}_{g, son}$, whose attributing marker M = 0, of $\Omega^k_{g, lv}$ and $\Omega^k_{g, rv}$. The amount of the $\Omega^{k+1}_{g, son}$ is $N^{k+1}_{g, son}$.

If $N^{k+1}_{g, son} = 0$

End the circulation.

End

For *j* = 0 to $N^{k+1}_{g, son}$

$G = G_j$, $G_j \in \Omega^{k+1}_{g, son}$, the connected areas of node G are A_G

According to formula (7) and (9), judge the attribution of nodes:

If $M_2 = 3$, $A_{lv} \leftarrow A_{lv} \cup A_G$, $\Omega_{g, lv} \leftarrow \Omega_{g, lv} + G$

If $M_2 = 4$, $A_{rv} \leftarrow A_{rv} \cup A_G$, $\Omega_{g, rv} \leftarrow \Omega_{g, rv} + G$

If $M_G = 8$, detect area $A_{fl, cn}$, which connects A^k_{lv} and A^k_{rv}. Removing $A_{fl, cn}$ from A_G , obtaining all connected areas $\{ A_{re, 1}, A_{re, 2}, \ldots\ldots \}$ from the remaining area and $VA_{re, h} \in \{ A_{re, 1}, A_{re, 2}, \ldots\ldots \}$. Calculating similarity d_1 between $A_{re, h}$ and A^k_{lv} and calculating similarity d_2 between $A_{re, h}$ and A^k_{rv} . Obtaining the attributing marker of $A_{re, h}$, M_2 .

If $M_2 = 1$, $A_{lv} \leftarrow A_{lv} \cup A_{re, h}$, $\Omega_{g, lv} \leftarrow \Omega_{g, lv} + G$

If $M_2 = 0$, $A_{rv} \leftarrow A_{rv} \cup A_{re, h}$, $\Omega_{g, rv} \leftarrow \Omega_{g, rv} + G$

End

$A_{all, lv} \leftarrow A_{all, lv} + A_{lv}$, $A_{all, rv} \leftarrow A_{all, rv} + A_{rv}$,

$\Omega_{all, lv} \leftarrow \Omega_{all, lv} + \Omega_{g, lv}$, $\Omega_{all, rv} \leftarrow \Omega_{all, rv} + \Omega_{g, rv}$

End

Output: the set of LV cavity nodes $\Omega_{all,\ lv}$ and the connected areas $A_{all,\ lv}$, the set of RV cavity nodes $\Omega_{all,\ rv}$ and the connected areas $A_{all,\ rv}$, attributing marker M.

Paralleled VSD detecting algorithm:

Input: the set of LV cavity nodes $\Omega_{all,\ lv}$ and the connected areas $A_{all,\ lv}$, the set of RV cavity nodes $\Omega_{all,\ rv}$ and the connected areas $A_{all,\ rv}$, the attributing marker M, the 3D graphical data trove Ω_g, the set of original CT images Is, the amount of original CT images N and the set of the cardiac cavities images Ip.

All set of paralleled VSD nodes $\Omega_{all,\ lk}$ and connected areas $A_{all,\ lk}$

For *i* = 0: N

According to the data structure Ω_g on frame *i*, obtain the node *G* whose M = 8

For G in Ω_g^i :

If $M_G = 8$

1) $\Omega_{lk} \leftarrow G$, $A_{lk} \leftarrow A_G - (A_{G,\ rv} \cup A_{G,\ lv})$

2) $\Omega_{all,\ lk} \leftarrow \Omega_{all,\ lk} \cup \Omega_{lk}$, $A_{all,\ lk} \leftarrow A_{all,\ lk} \cup A_{lk}$

End

End

Output：all sets of paralleled VSD nodes $\Omega_{all,\ lk}$ and the connected areas $A_{all,\ lk}$

5.3 Detection of ventricular cavity on images with oblique VSD

To recognize the targeted ventricular cavity on the images with oblique VSD, the transition

nodes from LV to RV (or RV to LV) G_{key} were found through node marker M, obtained using formulae (7) and (9). Part of the transition node belonging to both the LV and RV was defined as the intersection area. The left side was the process of the intersection area changing from LV to RV, and the right side was the process of the intersection area changing from RV to LV (Supplementary Figure 4c). The yellow circle indicates a transition node, and the number in the circle indicates the proportion of the intersection area to the entire connected area of the transition node. V1 indicates the search direction of the transition node on the LV structure map $\Omega_{all, lv}$, and v2 indicates the search direction of the transition node on the RV structure map $\Omega_{all, rv}$. When searching in the direction of v1 and v2, the search process ended when the proportion of the intersection area to the whole connected area of the transition node r_{vsd} was less than the threshold value T^{r}_{low}. The intersectional area of the transition node G_{key} was the intersection of the parent node and the son node A_{vsd} when the LV and RV structure maps were searched along the v1 and v2 directions. When the proportion of the intersection area to the whole connected area of transition node $r_{vsd} > T^{r}_{up}$, the entire connected area of node G acted as a new intersection area, and the new intersection area represented VSD.

$$A_{vsd} = A_G \tag{11}$$

Oblique VSD detecting algorithm:

Input：the set of LV cavity nodes $\Omega_{all, lv}$ and the connected areas $A_{all, lv}$, the set of RV cavity nodes $\Omega_{all, rv}$ and the connected areas $A_{all, rv}$, the attributing marker M, the 3D graphical data trove Ω_g, the set of original CT images Is, the amount of original CT images N, and the set of the cardiac cavities images Ip.

All set of oblique VSD nodes $\Omega_{all, vsd}$ and connected areas $A_{all, vsd}$

For $i = 0: N$

According to the 3D graphical data trove Ω_g on frame i, obtain the node G whose son nodes G_{son} or parent node G_{parent} belong to a different ventricular cavity, compared with node G.

For G in Ω_g^i :

If $G_{parent} \in \Omega_{all, lv}$, $G_{son} \in \Omega_{all, rv}$ or $G_{parent} \in \Omega_{all, rv}$, $G_{son} \in \Omega_{all, lv}$

1) Searching $\Omega_{all, lv}$ in the direction of V1 and V2; searching $\Omega_{all, rv}$ in the direction of V1 and V2.
2) When the proportion of the oblique VSD area to the whole connected area is < T_{low}^r, end the search and obtain the set of oblique VSD nodes Ω_{vsd} and connected areas A_{vsd} .
3) $\Omega_{all, vsd} \leftarrow \Omega_{all, vsd} \cup \Omega_{vsd}$, $A_{all, vsd} \leftarrow A_{all, vsd} \cup A_{vsd}$

End

End

Output：all sets of oblique VSD nodes $\Omega_{all, vsd}$ and connected areas $A_{all, vsd}$.

6. Detection of the great vessel cavity region

The great arterial cavity was detected after the left and right ventricular cavities were detected. The connected area of the aorta cavity and pulmonary arterial cavity on frame k were assumed to be A_{ao}^k and A_{pa}^k, respectively, and the corresponding nodes were assumed to be $\Omega_{g, ao}^k$ and $\Omega_{g, pa}^k$, respectively. Similar concepts of the ventricular cavity recognition algorithm were used to

obtain the node set $\Omega_{all, ao}$, the connected areas set $A_{all, ao}$ of the aorta cavity, the node set $\Omega_{all, pa}$ and the connected areas set $A_{all, pa}$ of the pulmonary arterial cavity (see Supplementary Method 5.1). If one great artery was spatially connected to one ventricle, the artery was directly attributed to that ventricle.

When the great artery was connected to the ventricle, a large number of repeated searches for ventricular cavity nodes might occur during the detection of the great artery cavity. Fewer son nodes were found in the great arterial cavity region compared with the ventricular cavity regions. To avoid repeated and meaningless searches, a constraint condition was set when the artery was searched forward:

$$\varepsilon=\begin{cases} 1, & N_{g,st}^{k} > N_{g,st}^{k0} + aN \\ 0, & N_{g,st}^{k} \leqslant N_{g,st}^{k0} + aN \end{cases} \tag{12}$$

st = ao represents the aorta, and st = pa represents the pulmonary artery. $N_{g, st}^{k0}$ represents the number of connected nodes on input frame *K0*, $N_{g, st}^{k}$ represents the number of connected nodes on input frame *k*. As a constant, aN represents the possibility of the maximum number of subareas of a great artery.

When ε = 0, keep searching forward; when ε = 1, end the searching process. After updating the 3D graphical data trove with the information of the ventricular cavity and the great arterial cavity was completed, because the semilunar valves or blood clots in the great arterial cavity might clog the cavity completely, the ventricular cavity and the great arterial cavity were not connected. Here, to determine the relationship between the ventricular cavity and the great arterial cavity, images (n = *cR*) after the left and RV node set were searched to find all the last connected areas of the left and RV cavity that had no son nodes. $A_{zero, lv}$ and

$A_{zero, rv}$, respectively, and searched frames (n = *aR*) ahead of the great arterial cavity node set to obtain all the first connected areas of the great arterial cavity $A_{zero, st}$. By observing the heart, the great arterial cavity was found to be connected to the nearer ventricular cavity. The shortest distances between $A_{zero, st}$ and $A_{zero, lv}$ and between $A_{zero, st}$ and $A_{zero, rv}$ were calculated to determine the connecting relationship between the marked great arterial cavities and ventricular cavities.

To calculate the distance, two connected areas A_1 and A_2 and $p_{1,i} \in A_1$ and $p_{2,j} \in A_2$ were assumed and the distance between the pixels of the two connected areas was defined as:

$$D_{p_{1,i}p_{2,j}} = \left\| p_{1,i} - p_{2,j} \right\|_2 \tag{13}$$

Then, the shortest distance between the two connected areas was calculated:

$$D = \min\left(\left\{ D_{p_{1,i}p_{2,j}} \mid p_{1.i} \in A_1, p_{2,j} \in A_2 \right\}\right) \tag{14}$$

According to formulae (13) and (14), the attribution of the great arterial cavities to the ventricular cavities was determined. Using the great arterial cavity extraction algorithm and inference of the great arteries algorithm, the LV and its related great arterial cavity node set $\Omega_{all, lv}$ and connected area set $A_{all, lv}$, as well as the RV and its related great arterial cavity node set $\Omega_{all, rv}$ and connected area set $A_{all, rv}$ were updated and obtained.

The great vessel cavity detecting algorithm:

Input: the aortic cavity region of the original image A_{ao}^{k0}, the pulmonary arterial cavity region of the original image A_{pa}^{k0}, the number of the original image *K0*, the 3D graphical data trove Ω_{g} , the set of original CT images Is, the amount of original CT images *N*, the set of the cardiac cavities images Ip, the set of aortic cavity nodes $\Omega_{all, ao}$ and the connected areas $A_{all, ao}$, the set of pulmonary arterial cavity nodes $\Omega_{all, pa}$ and the connected areas $A_{all, pa}$.

1) According to aortic and pulmonary arterial cavity region A_{ao}^{k0} and A_{pa}^{k0} on frame *k0*, obtain corresponding nodes $\Omega_{g, ao}^{k0}$ and $\Omega_{g, pa}^{k0}$,

$A_{all, ao} \leftarrow A_{all, ao} + A_{ao}^{k0}$, $A_{all, pa} \leftarrow A_{all, pa} + A_{pa}^{k0}$,

$\Omega_{all, ao} \leftarrow \Omega_{all, ao} + \Omega_{g, ao}^{k0}$, $\Omega_{all, pa} \leftarrow \Omega_{all, pa} + \Omega_{g, pa}^{k0}$

$A_{ao} \leftarrow A_{ao}^{k0}$, $A_{pa} \leftarrow A_{pa}^{k0}$, $\Omega_{g, ao} \leftarrow \Omega_{g, ao}^{k0}$, $\Omega_{g, pa} \leftarrow \Omega_{g, pa}^{k0}$

2) Search forward *sR0* frames from frame *k0*.

For $k = k0$ to sR0

$A_{ao}^{k} \leftarrow A_{ao}$, $A_{pa}^{k} \leftarrow A_{pa}$, $\Omega_{g, ao}^{k} \leftarrow \Omega_{g, ao}$, $\Omega_{g, pa}^{k} \leftarrow \Omega_{g, pa}$

$A_{ao} = O$, $A_{pa} = O$, $\Omega_{g, ao} = O$, $\Omega_{g,pa} = O$

According to 3D graphical data trove Ω_{g}, obtain parent nodes $\Omega_{g, parent}^{k-1}$ of $\Omega_{g, ao}^{k}$ and $\Omega_{g, pa}^{k}$. The amount of the $\Omega_{g, parent}^{k-1}$ is $N_{g, parent}^{k-1}$.

If $N_{g, parent}^{k-1} = 0$ and $N_{g, parent}^{k-1} > N(\Omega_{g, ao}^{k0}) + aN$

End circulation.

End

For $j = 0$ to $N_{g, parent}^{k-1}$

$G = G_j$, $G_j \in \Omega_{g, parent}^{k-1}$, the connected areas of node G are A_G

According to the similarity between node *G* and A^{k}_{ao} and the similarity between node *G* and A^{k}_{pa} , judge the attribution of node *G*:

If $M_2 = 5$, $A_{ao} \leftarrow A_{ao} \cup A_{G}$, $\Omega_{g, ao} \leftarrow \Omega_{g, ao} + G$

If $M_2 = 6$, $A_{pa} \leftarrow A_{pa} \cup A_{G}$, $\Omega_{g, pa} \leftarrow \Omega_{g, pa} + G$

End

$A_{all, ao} \leftarrow A_{all, ao} + A_{ao}$, $A_{all, pa} \leftarrow A_{all, pa} + A_{pa}$,

$\Omega_{all, ao} \leftarrow \Omega_{all, ao} + \Omega_{g, ao}$, $\Omega_{all, pa} \leftarrow \Omega_{all, pa} + \Omega_{g, pa}$

End

3) Search backward from frame *k0*.

$A_{ao} \leftarrow A^{k0}_{ao}$, $A_{pa} \leftarrow A^{k0}_{pa}$,$\Omega_{g, ao} \leftarrow \Omega^{k0}_{g, ao}$, $\Omega_{g, pa} \leftarrow \Omega^{k0}_{g, pa}$

For $k = k0$ to N

$A^{k}_{ao} \leftarrow A_{ao}$, $A^{k}_{pa} \leftarrow A_{pa}$, $\Omega^{k}_{g, ao} \leftarrow \Omega_{g, ao}$, $\Omega^{k}_{g, pa} \leftarrow \Omega_{g, pa}$

$A_{ao} = O$, $A_{pa} = O$, $\Omega_{g, ao} = O$, $\Omega_{g,pa} = O$

According to the 3D graphical data trove Ω_{g}, obtain son nodes $\Omega^{k+1}_{g, son}$ of $\Omega^{k}_{g, ao}$ and $\Omega^{k}_{g, pa}$. The amount of the $\Omega^{k+1}_{g, son}$ is $N^{k+1}_{g, son}$.

If $N^{k+1}_{g, son} = 0$

End circulation.

End

For *j* = 0 to $N^{k+1}_{g, son}$

$G = G_{j}$, $G_{j} \in \Omega^{k+1}_{g, son}$, the connected areas of node G are A_{G}

According to the similarity between node *G* and A^{k}_{ao} and the similarity between node *G* and A^{k}_{pa} , judge the attribution of node *G*:

If $M_2 = 5$, $A_{ao} \leftarrow A_{ao} \cup A_G$, $\Omega_{g, ao} \leftarrow \Omega_{g, ao} + G$

If $M_2 = 6$, $A_{pa} \leftarrow A_{pa} \cup A_G$, $\Omega_{g, pa} \leftarrow \Omega_{g, pa} + G$

End

$A_{all, ao} \leftarrow A_{all, ao} + A_{ao}$, $A_{all, pa} \leftarrow A_{all, pa} + A_{pa}$,

$\Omega_{all, ao} \leftarrow \Omega_{all, ao} + \Omega_{g, ao}$, $\Omega_{all, pa} \leftarrow \Omega_{all, pa} + \Omega_{g, pa}$

End

Output: the set of aortic cavity nodes $\Omega_{all, ao}$ and the connected areas $A_{all, ao}$, the set of pulmonary arterial cavity nodes $\Omega_{all, pa}$ and the connected areas $A_{all, pa}$, attributing marker M.

Determining the connecting relationship of ventricles and the great vessels

Input: the set of LV cavity nodes $\Omega_{all, lv}$ and the connected areas $A_{all, lv}$, the set of RV cavity nodes $\Omega_{all, rv}$ and the connected areas $A_{all, rv}$, the set of aortic cavity nodes $\Omega_{all, ao}$ and the connected areas $A_{all, ao}$, the set of pulmonary arterial cavity nodes $\Omega_{all, pa}$ and the connected areas $A_{all, pa}$, attributing marker M.

Obtain the first and last images of $\Omega_{all, lv}$, $\Omega_{all, rv}$, $\Omega_{all, ao}$ and $\Omega_{all, pa}$: [$F_{s, lv}$, $F_{e, lv}$], [$F_{s, rv}$, $F_{e, rv}$], [$F_{s, ao}$, $F_{e, ao}$] and [$F_{s, pa}$, $F_{e, pa}$]

Attribution of the great arteries

1) Determining whether there are nodes belonging to both the aortic cavity and LV (or RV). If the ventricular cavity and aortic cavity share no node, make Y_{ao} = 1. Attributing the aortic cavity to the ventricular cavity near to the cavity and making Y_{ao} = 0.

If $F_{s,ao} > F_{e, lv}$ and $\Omega_{all,lv}^{F_{e,lv}} \bigcap \Omega_{all,ao}^{F_{s,ao}} \neq O$

The aortic cavity originates from the LV cavity

$\Omega_{all, lv} \leftarrow \Omega_{all, lv} \cup \Omega_{all, ao}$, $A_{all, lv} \leftarrow A_{all, lv} \cup A_{all, ao}$

Elseif $F_{s,ao} > F_{e, rv}$ and $\Omega_{all,rv}^{F_{e,rv}} \bigcap \Omega_{all,ao}^{F_{s,ao}} \neq O$

The aortic cavity originates from the RV cavity

$\Omega_{all, rv} \leftarrow \Omega_{all, rv} \cup \Omega_{all, ao}$, $A_{all, rv} \leftarrow A_{all, rv} \cup A_{all, ao}$

End

2) Determining whether there are nodes belonging to both the pulmonary arterial cavity and LV (or RV). If the ventricular cavity and pulmonary arterial cavity share no node, make Y_{pa} = 1. Attributing the pulmonary arterial cavity to the ventricular cavity near to the cavity and making Y_{pa} = 0.

If $F_{s, pa} > F_{e, lv}$ and $\Omega_{all,lv}^{F_{e,lv}} \bigcap \Omega_{all,pa}^{F_{s,pa}} \neq O$

The aortic cavity originates from the LV cavity

$\Omega_{all, lv} \leftarrow \Omega_{all, lv} \cup \Omega_{all, pa}$, $A_{all, lv} \leftarrow A_{all, lv} \cup A_{all, pa}$

Elseif $F_{s, pa} > F_{e, rv}$ and $\Omega_{all,rv}^{F_{e,rv}} \bigcap \Omega_{all,pa}^{F_{s,pa}} \neq O$

The aortic cavity originates from the RV cavity

$\Omega_{all, rv} \leftarrow \Omega_{all, rv} \cup \Omega_{all, pa}$, $A_{all, rv} \leftarrow A_{all, rv} \cup A_{all, pa}$

End

3) If $Y_{ao} = 1$ or $Y_{pa} = 1$, searching frames (n = *cR*) backward ventricular cavity nodes to find all nodes of ventricular cavity containing no son node.

Searching backward frames (n = *cR*) of LV cavity nodes $\Omega_{all, lv}$ to find all connected areas $A_{zero, lv}$ containing no son node.

For *k* = $F_{e, lv}$ - cR to $F_{e,lv}$

$\forall G \in \Omega^{k}_{all,lv}$, determining the number of son nodes of node *G*, expressed as $N_{G, son}$

If $N_{G, son}$ =0

$A_{zero, lv} \leftarrow A_{zero, lv} \cup A_{G}$

End

End

Searching backward frames (n = *cR*) of RV cavity nodes $\Omega_{all, rv}$ to find all connected areas $A_{zero, rv}$ without a son node.

For *k*= $F_{e, rv}$ - cR to $F_{e,rv}$

$\forall G \in \Omega^{k}_{all,rv}$, determining the amount of son nodes of node *G*, expressed as $N_{G, son}$

If $N_{G, son}$ =0

$A_{zero, rv} \leftarrow A_{zero, rv} \cup A_{G}$

End

End

4) If Y_{ao} = 1, searching forward frames (n = *aR*) of the set of aortic cavity nodes $\Omega_{all,\ ao}$ to find all connected areas $A_{zero,\ ao}$ without a parent node. Calculating the shortest distance between $A_{zero,\ ao}$ and $A_{zero,\ lv}$. Calculating the shortest distance between $A_{zero,\ ao}$ and $A_{zero,\ rv}$. Judging the origination of the aortic cavity.

For $k = F_{s,\ ao}$ to $F_{s,\ ao} + aR$

$\forall G \in \Omega^{k}_{all,ao}$, determining the amount of parent nodes of node *G*, expressed as $N_{G,\ parent}$

If $N_{G,\ parent} = 0$

$A_{zero,\ ao} \leftarrow A_{zero,\ ao} \cup A_{G}$

End

End

According to formulae (13) and (14), calculating the shortest distance D_1 between $A_{zero,\ ao}$ and $A_{zero,\ lv}$ and calculating the shortest distance D_2 between $A_{zero,\ ao}$ and $A_{zero,\ rv}$.

If $D_1 > D_2$

The aortic cavity originates from the LV cavity

$\Omega_{all,\ lv} \leftarrow \Omega_{all,\ lv} \cup \Omega_{all,\ ao}$, $A_{all,\ lv} \leftarrow A_{all,\ lv} \cup A_{all,\ ao}$

Otherwise

The aortic cavity originates from the RV cavity

$\Omega_{all,\ rv} \leftarrow \Omega_{all,\ rv} \cup \Omega_{all,\ ao}$, $A_{all,\ rv} \leftarrow A_{all,\ rv} \cup A_{all,\ ao}$

End

5) If Y_{pa} = 1, searching forward frames (n = *aR*) of the set of pulmonary arterial cavity nodes $\Omega_{all, pa}$ to find all connected areas $A_{zero, pa}$ without a parent node. Calculating the shortest distance between $A_{zero, pa}$ and $A_{zero, lv}$. Calculating the shortest distance between $A_{zero, pa}$ and $A_{zero, rv}$. Judging the origination of the pulmonary arterial cavity.

For $k = F_{s, pa}$ to $F_{s, pa} + aR$

$\forall G \in \Omega^{k}_{all,pa}$, determining the number of parent nodes of node *G*, expressed as $N_{G, parent}$

If $N_{G, parent} = 0$

$A_{zero, pa} \leftarrow A_{zero, pa} \cup A_{G}$

End

End

According to formulae (13) and (14), calculating the shortest distance D_1 between $A_{zero, pa}$ and $A_{zero, lv}$ and calculating the shortest distance D_2 between $A_{zero, pa}$ and $A_{zero, rv}$.

If $D_1 > D_2$

The pulmonary arterial cavity originates from the LV cavity

$$\Omega_{all, lv} \leftarrow \Omega_{all, lv} \cup \Omega_{all, pa},\ A_{all, lv} \leftarrow A_{all, lv} \cup A_{all, pa}$$

Otherwise

The pulmonary arterial cavity originates from the LV cavity

$$\Omega_{all, rv} \leftarrow \Omega_{all, rv} \cup \Omega_{all, pa},\ A_{all, rv} \leftarrow A_{all, rv} \cup A_{all, pa}$$

End

Output: the updated set of LV cavity nodes $\Omega_{all, lv}$ and the connected areas $A_{all, lv}$, the updated set of RV cavity nodes $\Omega_{all, rv}$ and the connected areas $A_{all, rv}$.

7. Optimization of filtered nodes (detailed cavities)

Before detecting the atrial region and acquiring the inference process from ventricular cavities

to the great arterial cavities, all connected areas less than the area-size threshold T_a were filtered to avoid the impact of small areas, as follows:

$$M_i^k = \begin{cases} 1, S_i^k < T_a \\ 0, S_i^k \geq T_a \end{cases} \tag{15}$$

S_i^k represents the size of the connected area of node *i* on frame *k*; M_i^k represents the attributing marker of node *i* on frame *k*; $M_i^k = 0$ indicates that node *k* was not processed; and $M_i^k = 1$ indicates that node *k* is filtered out. In addition, in the forward or backward search for ventricular and great arterial cavities, some nodes without a parent node or son node were neglected. When searching from frame *G1* in the V1 direction (Supplementary Figure 3c), the red node did not have a parent node and was neglected in the search for ventricular and great arterial cavities. When searching from frame *G3* in the V2 direction (Supplementary Figure 3c), the orange node did not have a son node and was neglected in the search for ventricular and great arterial cavities. Therefore, after obtaining the whole inference process from ventricular cavities to the great arterial cavities, these neglected or filtered connected areas were reassessed. Some regions had not been searched in the forward or backward search process for ventricular and great arterial cavities and should be classified into a corresponding inference process.

Optimization of filtered nodes:

Input: the updated set of LV cavity nodes $\Omega_{all, lv}$ and the connected areas $A_{all, lv}$, the updated set of RV cavity nodes $\Omega_{all, rv}$ and the connected areas $A_{all, rv}$, the attributing marker M, the 3D graphical data trove Ω_g, the set of original CT images Is, the amount of original CT images *N* and the set of the cardiac cavities images Ip.

1) Obtaining the first and last images of $\Omega_{all, lv}$ and $\Omega_{all, rv}$: [$F_{s, lv}$, $F_{e, lv}$] and [$F_{s, rv}$, $F_{e, rv}$]

   F0 = min ($F_{s, lv}$, $F_{s, rv}$), F1 = max ($F_{e, lv}$, $F_{e, rv}$)

2) Searching the ventricular cavity nodes backward

   For *i* = F0: F1

   Searching the updated set of LV cavity nodes $\Omega_{all, lv}$ on frame *i* to find all son nodes in which the attributing marker M = 0 or M = 1. Determining whether the son node belongs to the LV cavity, according to the similarity between the son node and LV cavity node on frame *i*. If the son node belongs to the LV cavity, adding the son node to $\Omega^{i+1}_{all, lv}$.

   Searching the updated set of RV cavity nodes $\Omega_{all, rv}$ on frame *i* to find all son nodes in which the attributing marker M = 0 or M = 1. Determining whether the son node belongs to the RV cavity, according to the similarity between the son node and RV cavity node on frame *i*. If the son node belongs to the RV cavity, adding the son node to $\Omega^{i+1}_{all, rv}$.

   End

3) Searching the ventricular cavity nodes forward

   For *i* = F1: F0

Searching the updated set of LV cavity nodes $\Omega_{all, lv}$ on frame *i* to find all parent nodes in which the attributing marker M = 0 or M = 1. Determining whether the parent node belongs to the LV cavity, according to the similarity between the parent node and LV cavity node on frame *i*. If the parent node belongs to the LV cavity, add the parent node to $\Omega^{i-1}_{all, lv}$.

Searching updated set of RV cavity nodes $\Omega_{all, rv}$ on frame *i* to find all parent nodes in which the attributing marker M = 0 or M = 1. Determining whether the parent node belongs to the RV cavity, according to the similarity between the parent node and RV cavity node on frame *i*. If the parent node belongs to the RV cavity, add the parent node to $\Omega^{i-1}_{all, rv}$.

End

Output: the updated set of LV cavity nodes $\Omega_{all, lv}$ and the connected areas $A_{all, lv}$, the updated set of RV cavity nodes $\Omega_{all, rv}$ and the connected areas $A_{all, rv}$.

Supplementary Table 1 Attributing markers of nodes in CT images

**Attribution of nodes**	**Attributing marker (M)**
Not processed	M=0
Filtered out	M=1
AC	M=2
LVC	M=3
RVC	M=4
Aortic cavity	M=5
Pulmonary arterial cavity	M=6
the AC and areas connected to AC	M=7
Nodes connected LVC with RVC	M=8
Undefined state	M=9

AC, atrial cavity; LVC, left ventricular cavity; RVC, right ventricular cavity.

Supplementary Movie 1 CACCT-detected ventricular and the great arterial cavities from E17.5 normal murine hearts.

CACCT detection shows the right ventricle connected to the pulmonary artery and the left ventricle to the aorta, which corresponds to the anatomy of the murine heart. Green line, right ventricle and pulmonary artery; blue line, left ventricle and aorta. n = 5 hearts.

Supplementary Movie 2 CACCT-detected cavity regions merged with manually marked regions from E17.5 normal murine hearts.

CACCT identifies that the right ventricle is connected to the pulmonary artery and the left ventricle to the aorta, which is consistent with the anatomy of the murine heart. Green line, right ventricle and pulmonary artery and aorta; blue line, left ventricle and aorta; red line, manually marked ventricular and great arterial cavities. n = 5 hearts.

Supplementary Movie 3 CACCT-generated 3D cardiac cavity images from E17.5 retinoic acid-induced hearts with transposition of the great arteries (TGA).

The transparent gray volume depicts the myocardium, generated by reconstruction of the CT images without image processing. The blue 3D volume represents the CACCT-detected right ventricle (RV) and aorta (Ao) originating from the RV. The red 3D volume represents the CACCT-detected left ventricle (LV) and pulmonary artery (PA) originating from the LV. The geometry (e.g., shape, position) corresponds to the TGA diagnosis. n = 4 hearts.

Supplementary Movie 4 CACCT-generated 3D cardiac cavity images from E17.5 retinoic acid-induced hearts with double-outlet right ventricle (DORV).

The transparent gray volume depicts the myocardium, generated by reconstruction of the CT images without image processing. The blue 3D volume represents the CACCT-detected right ventricle (RV), aorta (Ao) and pulmonary artery (PA). Two great arteries both originated from the RV. The red 3D volume represents the CACCT-detected left ventricle (LV). The geometry

(e.g., shape, position) corresponds to the DORV diagnosis. n = 5 hearts.

Supplementary Movie 5 CACCT-detected cavity regions from E17.5 retinoic acid-induced murine hearts with transposition of the great arteries (TGA).

CACCT identifies that the right ventricle is connected to the aorta and the left ventricle to the pulmonary artery, which is consistent with the diagnosis made by senior fetal cardiologists and the clinical definition of TGA. Green line, right ventricle and aorta; blue line, left ventricle and pulmonary artery. n = 4 hearts.

Supplementary Movie 6 CACCT-detected cavity regions from E17.5 retinoic acid-induced (RA-induced) murine hearts with double-outlet right ventricle (DORV).

CACCT identifies that the right ventricle is connected to the pulmonary artery aorta, which is consistent with the diagnosis made by senior fetal cardiologists and the clinical definition of DORV. Green line, right ventricle, pulmonary artery and aorta; blue line, left ventricle. n = 5 hearts.

Supplementary Movie 7 CACCT-detected VSD types I and II and corrected segmented ventricular cavities from E17.5 retinoic acid-induced hearts.

CACCT detection shows that paralleled VSD connects the right ventricle with the left ventricle on one section. oblique VSD obliquely intersects the transverse sections and connects only one or no ventricles on one section. Green line, right ventricle, pulmonary artery and aorta; blue line, left ventricle; dark pink area, paralleled VSD; light pink areas; oblique VSD. n = 9 hearts.

Supplementary Movie 8 Manually marked ventricular and great arterial cavities in transverse sections of the CT image dataset obtained from E17.5 normal murine hearts.

n = 5 hearts.

Supplementary Movie 9 3D cardiac cavity images generated by CACCT from E17.5 normal murine hearts.

The transparent gray volume depicts the myocardium, generated by CT image reconstruction without image processing. The blue 3D volume represents the CACCT-detected right ventricle (RV) and pulmonary artery (PA) originating from the RV. The red 3D volume represents the CACCT-detected left ventricle (LV) and aorta (Ao) originating from the LV. The geometry (e.g., shape, position) corresponds to the anatomy of the murine ventricles and the great arteries. n = 5 hearts.
